# A novel evolutionary conserved mechanism of RNA stability regulates synexpression of primordial germ cell-specific genes prior to the sex-determination stage in medaka

**DOI:** 10.1371/journal.pbio.3000185

**Published:** 2019-04-04

**Authors:** Amaury Herpin, Cornelia Schmidt, Susanne Kneitz, Clara Gobé, Martina Regensburger, Aurélie Le Cam, Jérome Montfort, Mateus C. Adolfi, Christina Lillesaar, Dagmar Wilhelm, Michael Kraeussling, Brigitte Mourot, Béatrice Porcon, Maëlle Pannetier, Eric Pailhoux, Laurence Ettwiller, Dirk Dolle, Yann Guiguen, Manfred Schartl

**Affiliations:** 1 INRA, UR1037 Fish Physiology and Genomics, Rennes, France; 2 University of Wuerzburg, Physiological Chemistry, Biocenter, Wuerzburg, Germany; 3 UMR BDR, INRA, ENVA, Université Paris Saclay, Jouy en Josas, France; 4 University of Melbourne, Department of Anatomy & Neuroscience, Parkville, Victoria, Australia; 5 University of Heidelberg, Centre for Organismal Studies (COS), Department of Developmental Biology, Heidelberg, Germany; 6 Comprehensive Cancer Center Mainfranken, University Hospital, Wuerzburg, Germany; 7 Hagler Institute for Advanced Study and Department of Biology, Texas A&M University, College Station, Texas, United States of America; University of Michigan, UNITED STATES

## Abstract

Dmrt1 is a highly conserved transcription factor, which is critically involved in regulation of gonad development of vertebrates. In medaka, a duplicate of dmrt1—acting as master sex-determining gene—has a tightly timely and spatially controlled gonadal expression pattern. In addition to transcriptional regulation, a sequence motif in the 3′ UTR (D3U-box) mediates transcript stability of dmrt1 mRNAs from medaka and other vertebrates. We show here that in medaka, two RNA-binding proteins with antagonizing properties target this D3U-box, promoting either RNA stabilization in germ cells or degradation in the soma. The D3U-box is also conserved in other germ-cell transcripts, making them responsive to the same RNA binding proteins. The evolutionary conservation of the D3U-box motif within *dmrt1* genes of metazoans—together with preserved expression patterns of the targeting RNA binding proteins in subsets of germ cells—suggest that this new mechanism for controlling RNA stability is not restricted to fishes but might also apply to other vertebrates.

## Abstract 2

In medaka a duplicate of dmrt1 acts as master sex-determining gene, with tightly controlled expression pattern in the gonads. This study shows that two RNA-binding proteins with antagonistic properties target a sequence motif in the 3’-UTR (D3U-box), promoting either RNA stabilization in germ cells or degradation in the soma.

## Author summary

The development of the gonads in vertebrates is mainly regulated by *dmrt1*, a master sex-determining gene that has a timely and spatially controlled gonadal expression pattern. In addition to transcriptional regulation, a sequence motif located in the 3′ UTR (D3U-box) mediates transcript stability of dmrt1 mRNAs. However, this regulation is complex, and the mechanisms are barely understood. Here, we analyse further dmrt1 regulation in the fish model medaka and show that two RNA-binding proteins with antagonizing properties target the D3U-box and promote either RNA stabilization in germ cells or degradation in the soma. The evolutionary conservation of the D3U-box motif within metazoan *dmrt1* genes—together with preserved expression patterns of the targeting RNA binding proteins in subsets of germ cells—suggest that this new mechanism for controlling RNA stability is not restricted to fishes but might also apply to other vertebrates.

## Introduction

The gonads of vertebrates are characterized by the intimate association of germ cells and supporting somatic cells [1–4]. The precursor cells of the soma are derived from the embryonic lateral plate mesoderm, whereas germ cells originate from the germline lineage [5–9]. To carry out their highly specialized biological functions, the somatic gonadal primordium and the germline cells together must establish timely regulated programs of gene expression [1,10,11].

The *mab-3*/*doublesex*/*dmrt1* gene orthologs are, among metazoans, the most evolutionary conserved key regulators of the earliest phases of gonad development. They control complex gene regulatory networks specifying male gonadal primordium development as well as gonadal maintenance [12–15]. Remarkably, besides being firmly anchored within the regulatory network at critical nodes, *dmrt1* genes were found to act as upstream male sex determiners in organisms as phylogenetically diverse as flatworm [16], water flea [17], frog [18], flatfish [19], birds [20] and medaka [21,22].

During the last decade, much has been learned about how *dmrt1* as the most versatile sex gene triggers and controls gonad development. In human, it is a critical dosage-sensitive sex-determining gene, such that haploinsufficiency leads to XY male-to-female sex reversal and infertility [23,24]. In mice, it is required for male gonadal differentiation of somatic and germ cells [25–27], although *Dmrt1* appears to be dispensable for primary sex determination [15]. Dmrt1 also plays the decisive role in maintaining the cellular identity of the adult testis, most obvious from the fact that its malfunction in adult mutant mice gonads leads to transdifferentiation of Sertoli to granulosa-like cells and feminization of a fully developed testis [27]. Consequently, the action range of Dmrt1 is not restricted to initiation of the male gonadal phenotype during early development but also contributes to the active suppression of the female networks via repression of two ‘anti-testis’ pathways, Foxl2 and Wnt family member 4 (WNT4)/β-catenin (see [28] for review).

In sex determination model fish medaka, male sex determination is implemented by a male-specific primordial germ cell (PGC) mitotic arrest due to the activity of a Y-chromosome–specific duplicate version of *dmrt1*, designated *dmrt1bY* [29]. In *dmrt1* knockout mice, germ cells fail to arrest mitosis [30]. Further work on dmrt1 has shown it to be a transcriptional gatekeeper controlling mitosis versus meiosis decision in male germ cells [26]. Thus, dmrt1 in mice and dmrt1bY in medaka appear to be regulators of germ cell proliferation.

Despite its well-characterized crucial functions for gonad development in many vertebrates, the mechanisms that regulate the complex temporal and spatial expression pattern and guarantee precise levels of dmrt1 transcripts are only barely understood. Diverse regulatory mechanisms have been occasionally reported. Indirect transcriptional regulation of *dmrt1* upon steroid treatments has been described in several fish species (see [13] for review). Gonadal dimorphic expression of *dmrt1* has been suggested to be possibly under the control of differential CpG methylation of its promoter in two different flatfish species [31,32]. Similarly, in the red-eared slider turtle (*Trachemys scripta*), DNA methylation dynamics accounting for dmrt1 sexual dimorphic expression are tightly correlated with temperature [33]. In vitro transcriptional regulation assays revealed that binding sites for Sp1, Egr1 [34], and Gata4 [35] factors, which are present in the promoters of many genes, are also involved in transcriptional regulation of the rat *dmrt1* gene. And finally, evidence was presented that microRNA 224 (miR-224) promotes differentiation of mouse spermatogonial stem cells via direct targeting of *dmrt1*, decreasing its expression in testes [36]. Certainly in the context of ‘indirect’ regulation, *dmrt1* is one of the most prominent examples.

In medaka, for which a functional duplicate of the autosomal *dmrt1a* gene on the Y chromosome—*dmrt1bY*—became the master regulator of male sex determination [22,37], transcriptional rewiring was brought about by exaptation of two transposable elements, *Izanagi* and *Rex1*, co-opted to act as silencers. These turn off the somatic and the germ cell–specific expressions of the *dmrt1bY* gene [38, 39]. Thus far, two factors, dmrt1 itself [38] and sox5 [39], were identified, which turn off *dmrt1bY* expression after it has fulfilled its function in the early developing gonad [38, 39].

We previously identified a 11-bp sequence motif in the 3′ UTR of dmrt1bY (D3U-box, for dmrt1 3′ UTR box). This motif confers stability to the mRNA in the developing embryonic gonad, whereas in other tissues, the transcript is rapidly degraded [40], indicating that a post-transcriptional regulation mechanism could play a role in germline expression of dmrt1 in medaka.

Here, we show that the dmrt1 11-nucleotide *cis*-regulatory D3U-Box motif is a target for two antagonizing RNA binding proteins, *O**ryzias* *l**atipes* CUG-binding protein (Ol-cug-bp) and *O**ryzias* *l**atipes* Bicoid Stability Factor (Ol-bsf)—also known as cugbp Elav-like family member (celf) and leucine rich pentatricopeptide repeat containing (lrpprc), respectively, in mammals. In *Drosophila*, the bicoid stability factor (bsf) has initially been shown to be involved in regulating the stability of *bicoid* transcripts during oogenesis through binding structures within the 3′ UTR of transcripts that resemble CUG hairpins [41]. Later, bsf was also reported to have a role in regulation of early zygotic genes by binding a short consensus sequence (CAGGUA) in the 5′ UTR of genes expressed in the early zygote [42]. Cug-bp is the human homolog of the *Xenopus* eden-bp, which was shown to bind to mRNAs, such as *c-mos*, that exhibit rapid deadenylation following fertilization of oocytes [43]. Previous studies of cug-bp function have focused mainly on the roles of this protein in regulating alternative splicing [44] and also on its ability to modulate translation of several mRNAs [45]. However, as cug-bp is able to functionally substitute for eden-bp to induce deadenylation in *Xenopus* oocyte extracts [46], it seems likely that it also plays a similar role in regulating poly(A) shortening in mammalian cells. Indeed, it was shown that cug-bp can interact with poly(a)-specific ribonuclease (PARN) deadenylase to promote deadenylation of its substrate RNAs [47].

We find that in medaka, the D3U-box is targeted by these two different RNA binding proteins, with Ol-cug-bp1 leading to dmrt1bY degradation unless Ol-bsf is present in germ cells. Moreover, this new mechanism of dmrt1 RNA stability appears to regulate also the abundance of other transcripts specifically expressed in PGCs.

## Results

### Specific enrichment of a conserved *cis*-regulatory motif (D3U-box) in vertebrate genomes

**Fig 1:**
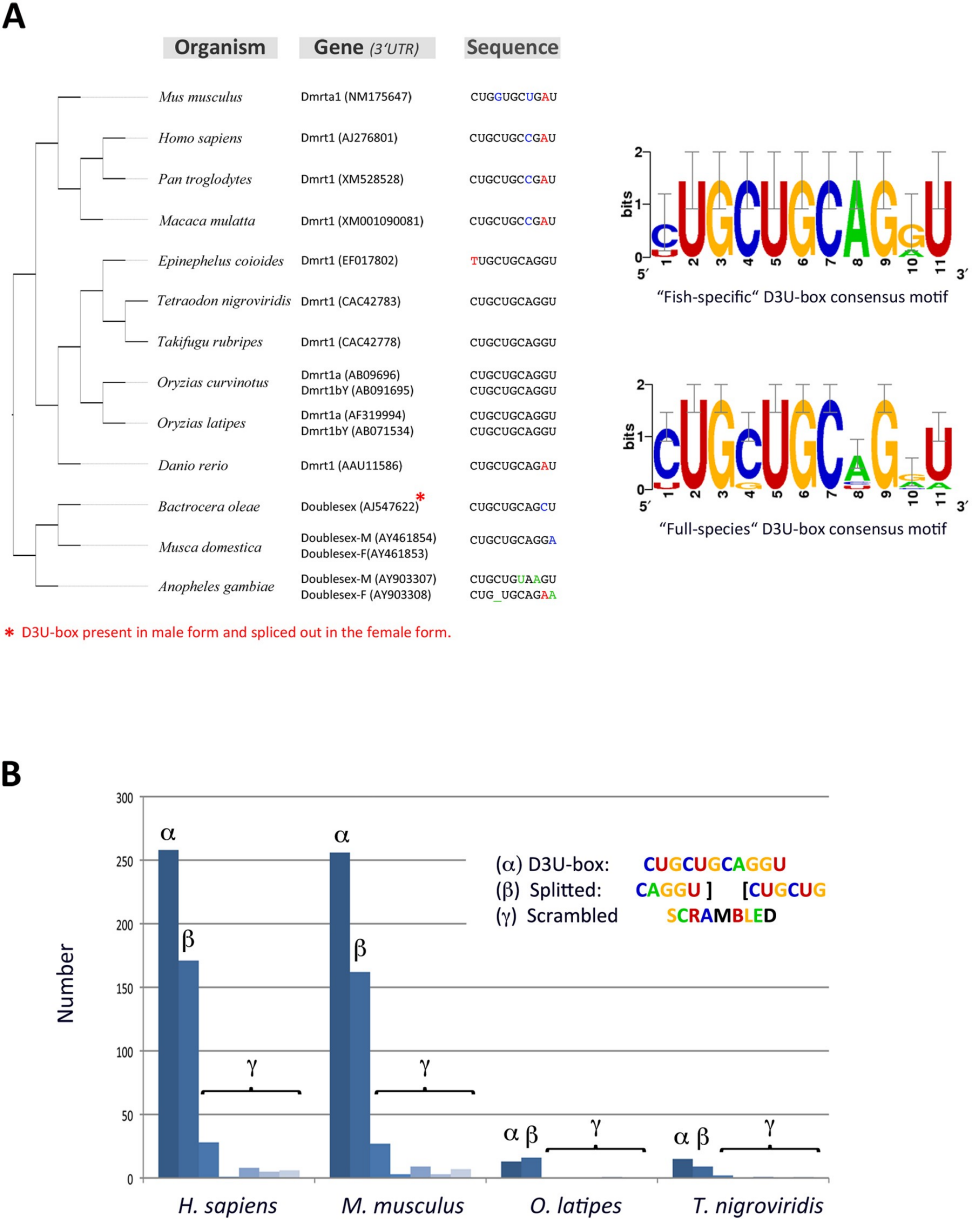
Conservation of the D3U-box motif from ecdysozoans to mammals. (A) Conservation of the D3U-box motif from *Drosophila* up to mammals. From the different D3U-box sequences among vertebrates, weight-position matrices were deduced and used for genome scans. (B) Occurrences of the D3U-box motif compared to control motifs within the 3′ UTR sequences of human, mouse, medaka, or the green spotted puffer (*Tetraodon nigroviridis*). For comparison, scrambled control motifs were used. Absolute values of the occurrences depend on the depth of annotation for every genome. Underlying data for (B) can be found in S1 Data. D3U-box, dmrt1 3′ UTR box.

In an initial analysis of dmrt1 post-transcriptional regulation [40], we found that an 11-bp long *cis*-regulatory motif in the D3U-box confers transcript stability in PGCs (Fig 1A). *In vitro* evidence was obtained that the D3U-box possibly mediates its function through protein binding [40]. Further on this sequence motif was found to be evolutionary conserved in *dmrt1* genes from flies to mammals (Fig 1A; [40]).

To investigate whether this motif is specific for the *dmrt1* genes or also present in other genes, we performed genome-wide searches for human, mouse, medaka, and the green spotted puffer (Fig 1B). The D3U-box was found in more than 250 3′ UTRs of genes from human and mice and is also abundant in medaka and green spotted puffer (Fig 1B). Notably, also, a ‘split’ version of the D3U-box (see Fig 1B) displayed specific enrichment in 3′ UTRs (Fig 1B), suggesting that the D3U-box motif might be articulated around two independent *cis*-regulatory sequences, hence putatively targeted by 2 different RNA binding proteins.

Medaka whole transcriptome scans (3′ UTR and coding sequences) using the vertebrate D3U-box consensus motif matrix (Fig 1A: 3′ UTR sequences; and S1 Fig: 3′ UTR and coding sequences) resulted in several hits, including tra2, sox10, misr2, dead end, and vasa (S1 Fig). Like dmrt1 [14,29,48,49], these RNAs are critically involved in germ cell development and maintenance in medaka and many other organisms ([36,50–55] and [56,57] for review).

Furthermore, bioinformatics analyses and literature searches [45,47] revealed that the D3U-box *cis*-regulatory motif is a putative target for 2 evolutionary conserved RNA binding proteins involved in either mRNA degradation or stabilization. These 2 proteins, Ol-cug-bp (also known as CELF in mammals) and Ol-bsf (also known as LRPPRC in mammals), have been shown to specifically recognize CUG repeats and the CAGGU(AG) motif, respectively, which constitute the D3U-box (see S2 Fig for phylogeny and synteny analysis of *Ol-bsf* and *Ol-cug-bps*).

### Ol-BSF and Ol-CUG-BP1 specifically bind to the D3U-box motif

**Fig 2:**
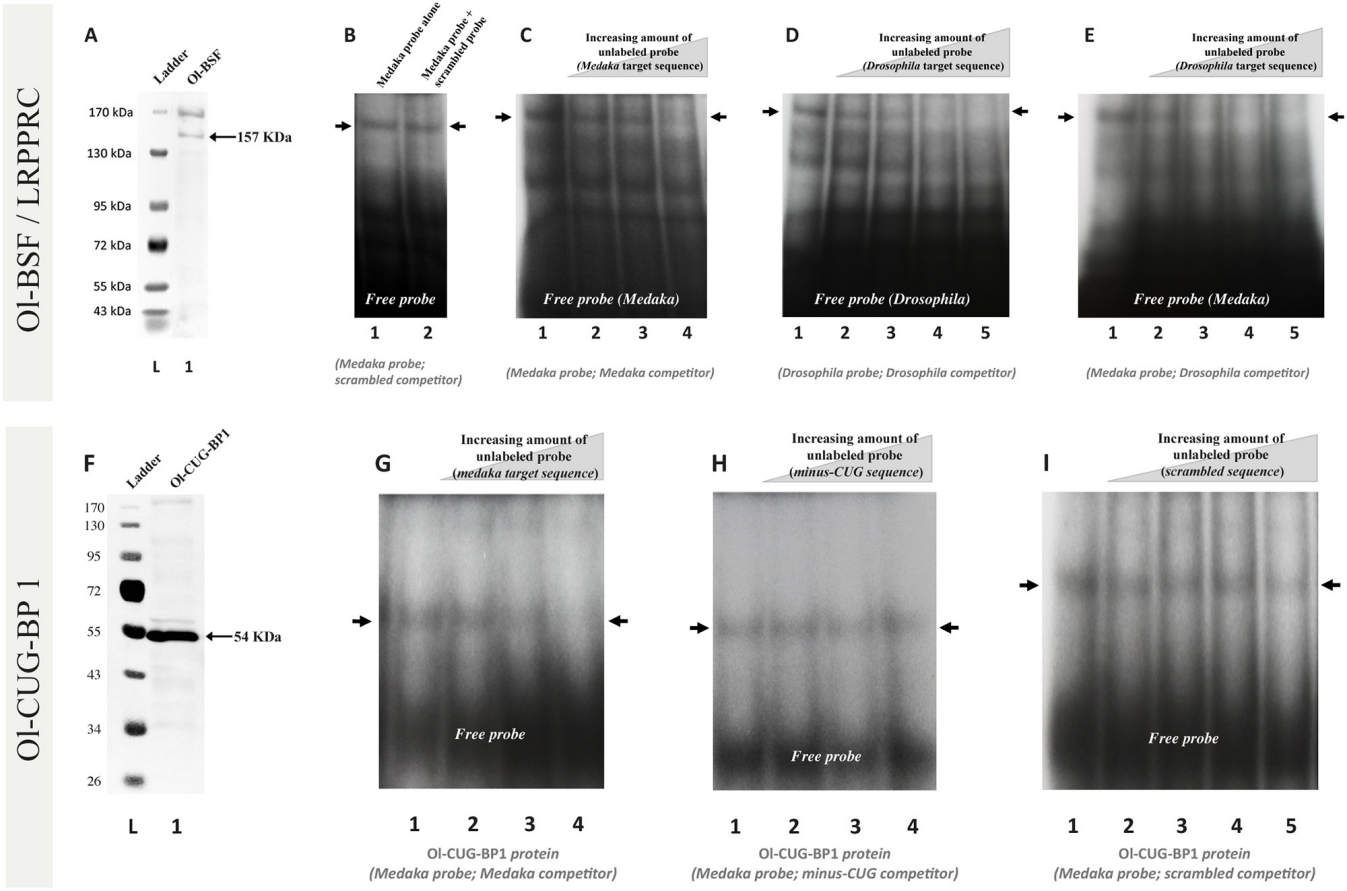
EMSA of in vitro–translated Ol-bsf, Ol-cug-bp1, and Ol-cug-bp2 proteins indicating interactions with the medaka D3U-box target sequence. (A and F) Recombinant protein production of FLAG-tagged versions of Ol-bsf and Ol-cug-bp1 and western blotting detection with an anti-FLAG antibody. (B–E and G–I) EMSA using the recombinant medaka Ol-bsf and Ol-cug-bp1 proteins (in vitro translated) and either the medaka D3U-box sequence (B, C, E, G, H, I), or the *Drosophila* D3U-box sequence (D) as radioactively labelled RNA probes. Increasing amounts of unlabelled probes (scrambled [B, I], medaka [C, G, K], *Drosophila* [D, E], minus CUG [H, L] target sequences) were used as competitors. (B–E and G–I) Apparent shifts are observed for the labelled RNA target probes with the Ol-bsf and Ol-cug-bp1 RNA-binding proteins, likely indicating direct interactions. Ratios of RNA probe to RNA competitor: (B, lane 2: 1/5; C, lane 2: 1/1, lane 3: 1/2, lane 4: 1/4; D, lane 2: 1/1, lane 3: 1/2, lane 4: 1/5, lane 5: 1/10; E, lane 2: 1/1, lane 3: 1/2, lane 4: 1/5, lane 5: 1/10; G, lane 2: 1/1, lane 3: 1/2, lane 4: 1/5; H, lane 2: 1/1, lane 3: 1/2, lane 4: 1/5; I, lane 2: 1/1, lane 3: 1/2, lane 4: 1/5, lane 5: 1/10). D3U-box, dmrt1 3′ UTR box; EMSA, electrophoretic mobility shift assay; lrpprc, leucine rich pentatricopeptide repeat containing; Ol-bsf, Oryzias latipes Bicoid Stability Factor; Ol-cug-bp, Oryzias latipes CUG-binding protein.

To confirm our bioinformatics prediction, Ol-bsf and Ol-cug-bp1 and 2 proteins were subjected to electrophoretic mobility shift assay (EMSA) using the D3U-box motif as target and different competitors (Fig 2). The *in vitro*–translated proteins (Fig 2A and 2F) were assayed for binding with radioactively labelled RNA probes. Using the D3U-box motif, mobility shifts were detected for the 2 proteins tested: Ol-bsf (Fig 2B to 2E) and Ol-cug-bp1 (Fig 2G to 2I), indicating that Ol-bsf as well as Ol-cug-bp1 are, in principle, able to bind the D3U-box in vitro. Binding specificities were confirmed by competition of the medaka D3U-box motif for Ol-bsf or Ol-cug-bp1 interactions with either a scrambled D3U-box–derived motif (Fig 2B and 2I) or a minus CUG repeat motif competitor (Fig 2H). The absence of any significant interference with the D3U-box binding indicated the specificity of the observed interactions (Fig 2B, 2I and 2H). Furthermore, competition experiments between radioactively and nonradioactively labelled D3U-boxes resulted in progressive loss of the apparent shifts (Fig 2C and 2G). Notably, a clearly visible shift was also observed when using the *Drosophila* D3U-box sequence together with the medaka Ol-bsf protein (Fig 2D). Altogether, these experiments suggest that the D3U-box is a preferential target for Ol-bsf and Ol-cug-bp1 binding. Of note, performing the very same set of experiments together with the Ol-cug-bp2 protein did not result in any convincing evidence for specific binding to the D3U-box.

### Ol-bsf and Ol-cug-bps antagonistically regulate the expression and stability of reporter constructs harbouring the D3U-box motif and of dmrt1bY transcripts

**Fig 3:**
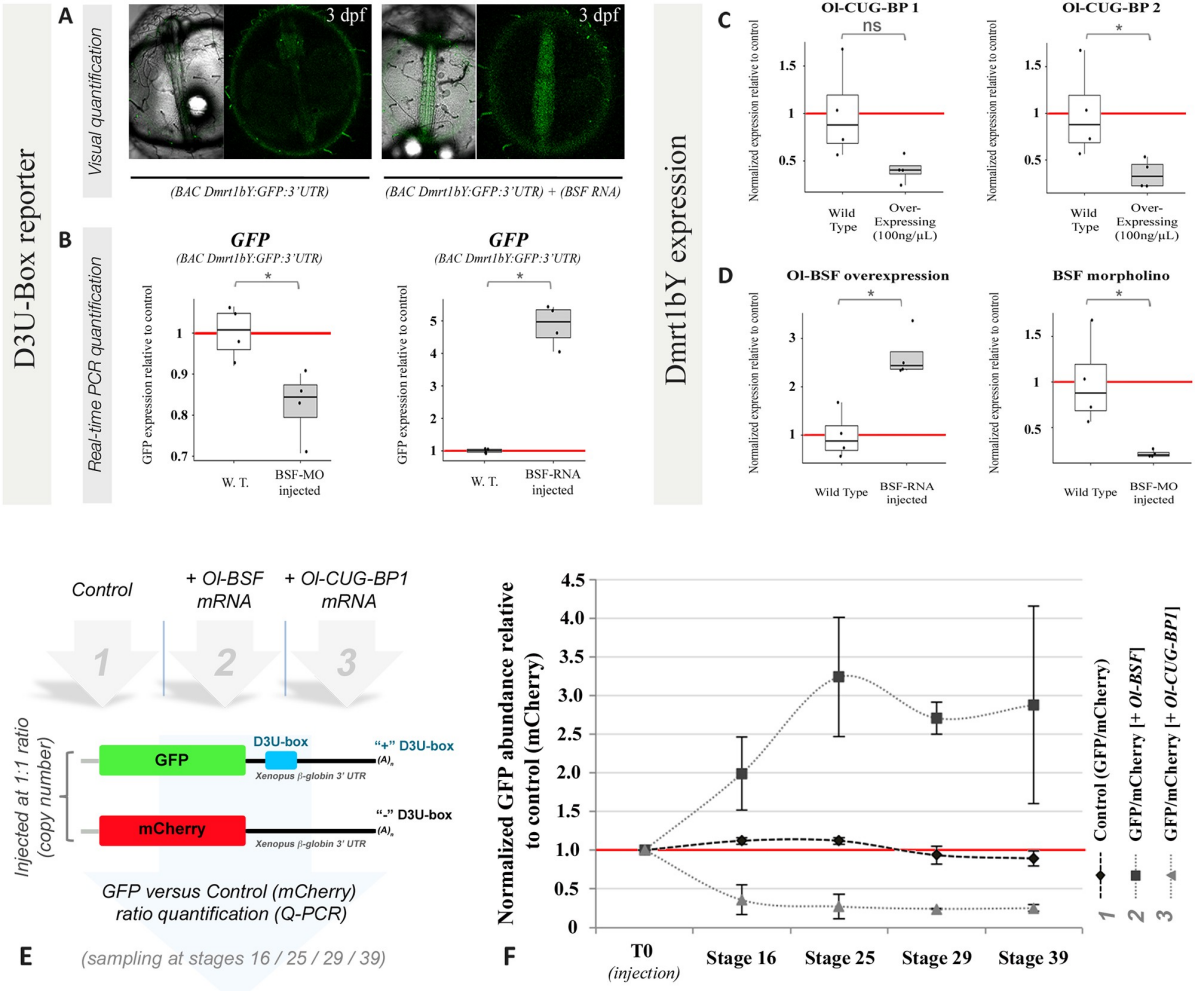
In vivo modulation of expression and mRNA stability of reporters and dmrt1bY transcripts via the D3U-box. (A and B) Effect of the modulation of the Ol-bsf expression on a GFP reporter for dmrt1bY expression. Injections of either Ol-bsf morpholino or capped mRNA result in the subsequent modulations of expression of a GFP:(dmrt1bY 3′ UTR) reporter/sensor construct in a transgenic line (BAC Dmrt1bY:GFP:3′UTR) as monitored by fluorescence (A) and RNA quantifications (B). Because endogenous GFP is lowly expressed already before experimental down-regulation, the more sensitive qPCR method was employed to monitor the morpholino effect (B). Dataset results of 4 independent reverse transcription reactions, resulting in 4 different batches of eggs obtained from different couples. Statistical significance has been assessed by mean of the Wilcoxon-Mann-Whitney test (*N* = 4). (C and D) Real-time qPCR determination of *dmrt1Y* abundances after Ol-cug-bp1 and Ol-cug-bp2 (C) or Ol-bsf (D) modulation of expression in embryos at stage 18 after injection of either capped mRNAs with the full Ol-bsf/Ol-cug-bps ORFs or a splice morpholino targeting the exon2-intron2 splice junction of *Ol-bsf* (S3 Fig). Dataset results of the analysis of 4 batches of eggs injected with either Ol-cug-bp1, Ol-cug-bp2, Ol-bsf, or BSF-MO. Statistical significance has been assessed by mean of the Wilcoxon-Mann-Whitney test (*N* = 4). *p ≤ 0.05, **p ≤ 0.01. (E and F) Real-time qPCR determination of the kinetics of RNA stability for D3U-box-containing reporters in presence or in absence of either Ol-cug-bp1 or Ol-bsf *coding mRNAs*. Datasets are results of 3 independent reverse transcription reactions, resulting from three different batches of injected eggs. Underlying data for (B, C, D, and F) can be found in S1 Data. BAC, bacterial artificial chromosome; D3U-box, *dmrt1* 3′ UTR box; GFP, green fluorescent protein; MO, Morpholino; ns, nonsignificant; Ol-BSF, *Oryzias latipes* Bicoid Stability Factor; Ol-CUG-BP, *Oryzias latipes* CUG-binding protein; ORF, open reading frame; qPCR, quantitative PCR; W.T., wild type.

To monitor a possible effect of Ol-bsf on regulation of the male sex-determination gene in medaka, we generated a dmrt1bY reporter line by introducing the green fluorescent protein (GFP) open reading frame (ORF) fused to the dmrt1bY 3′UTR (including the D3U-box) into exon 1 of a bacterial artificial chromosome (BAC) clone containing the *dmrt1bY* gene and flanking regions (Fig 3A and 3B). The recombined BAC was then used for establishing a stable transgenic line in which GFP expression most reliably indicates endogenous *dmrt1bY* expression [48,58,59]. Expression of dmrt1bY is highly dynamic during primordial gonad formation, progressively switching from germ cell expression only between stages 26 (1.25 dpf) and 29 (3.1 dpf) to an exclusive somatic expression from stages 33/34 (5 dpf) up to hatching (9 dpf) when the gonad is formed [39,59]. After injection of the Ol-bsf morpholino (see S3 Fig for validation of the morpholino), we found a significant reduction of GFP expression (Fig 3B) and, after Ol-bsf overexpression, a strong increase of the reporter at both mRNA and protein (fluorescence) levels (Fig 3A and 3B).

Next, to obtain a more physiological readout of the role(s) of Ol-bsf and Ol-cug-bps for RNA stability in vivo, the relative abundances of endogenous dmrt1bY transcripts were monitored after modulation of Ol-bsf/Ol-cug-bp1/Ol-cug-bp2 expression in medaka embryos (Fig 3C and 3D). First, we checked for changes in dmrt1bY transcript levels after overexpression of the 2 medaka Ol-cug-bp ohnologs (Ol-cug-bp1 and Ol-cug-bp2, Fig 3C). This resulted in decreased dmrt1bY mRNA (Fig 3C). Second, the relative abundances of dmrt1bY transcripts were recorded after either overexpression or morpholino knockdown of Ol-bsf in medaka embryos (Fig 3D). It revealed that higher Ol-bsf expression correlates with an increased abundance, while lowering Ol-bsf expression resulted in a reduction of dmrt1bY transcripts (Fig 3D). In vivo, D3U-box–induced modulation of RNA stability was further investigated in embryos injected with either control RNAs or RNAs harbouring the D3U-box (Fig 3E). Ratios between control and D3U-box–containing mRNAs were then quantified in absence or in presence of either l-cug-bp1 or Ol-bsf mRNAs (Fig 3F). It revealed that, over time, overexpression of Ol-bsf correlates with an increased stabilization of the D3U-box–containing mRNAs, while overexpression of Ol-cug-bp1 correlates with a decreased stabilization of the D3U-box–containing mRNAs (Fig 3F).

### Expression of Ol-cug-bp1, Ol-cug-bp2, and Ol-bsf during embryonic development and in adult tissues

During the embryonic developmental period, both Ol-cug-bp ohnologs display complementary patterns of expression. Ol-cug-bp1 is expressed at early stages and Ol-cug-bp2 only later when dmrt1bY transcripts appear in the germ cells around stage 25 (S4A and S4C Fig). In adult tissues, Ol-cug-bp1 and Ol-cug-bp2 are expressed at high levels in brain and gonads (S4B and S4D Fig), while Ol-cug-bp2 is additionally expressed in eyes, muscles, and skin (S4D Fig). Of note, both ohnologs are always more expressed in testes compared to ovaries (S4B and S4D Fig). Expression profiling of Ol-bsf revealed that in adult medaka, it is ubiquitously expressed in all adult tissues, with particular high levels in gonads of both sexes (S4E and S4F Fig).

### Ol-bsf is specifically expressed in the germ cells during early gonad primordium formation with correlated levels of expression between Ol-bsf and dmrt1bY at hatching stage

**Fig 4:**
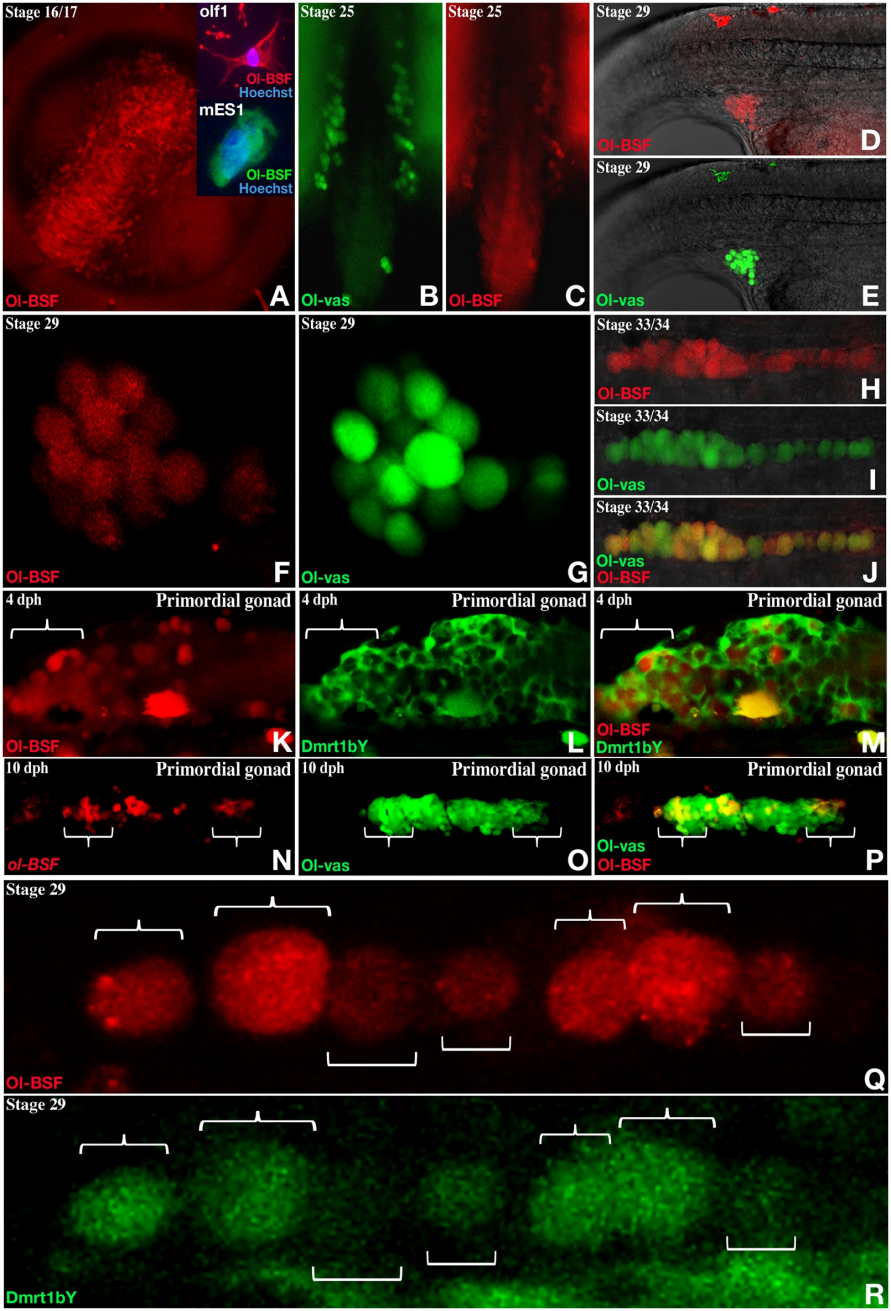
Embryonic expression of medaka Ol-bsf and correlated expression dynamics together with dmrt1bY during gonadal primordium formation. (A to R) Medaka Ol-bsf expression investigated by fluorescence using a transgenic reporter line for which a 732-bp *Ol-bsf* promoter fragment (up to the next upstream gene) drives the expression of the mCherry. Medaka Ol-vas and dmrt1bY expressions were investigated by fluorescence using previously described transgenic reporter lines ([11] and [61], respectively). (A) During neurulation (stages 16/17), Ol-bsf is ubiquitously expressed. (A insert) Cytoplasmic localisation of Ol-bsf after transient transfection of a 3-times FLAG version of Ol-bsf in a medaka embryonic stem cell line and immunohistochemistry. (B to P) From stage 25 on, Ol-bsf expression is restricted to the germ cells and colocalize with the germ cell marker Ol-vas. (K to P) Four-dph expression of Ol-bsf is apparently heterogeneous within the population of PGCs while dmrt1bY is now expressed in the surrounding somatic cells of the primordial gonad (K to M). (Q and R) By stage 29, Ol-bsf and dmrt1bY are both expressed in the germ cells. Variations within the respective levels of Ol-bsf and dmrt1bY are clearly observable amongst different germ cells although always correlated between the 2 fluorescences (curly brackets compared to square brackets). dph, days post hatching; MES-1, medaka embryonic stem cells; Ol-BSF, Oryzias latipes Bicoid Stability Factor; olf1, *Oryzias latipes* fibroblast-1; PGC, primordial germ cell.

**Fig 5:**
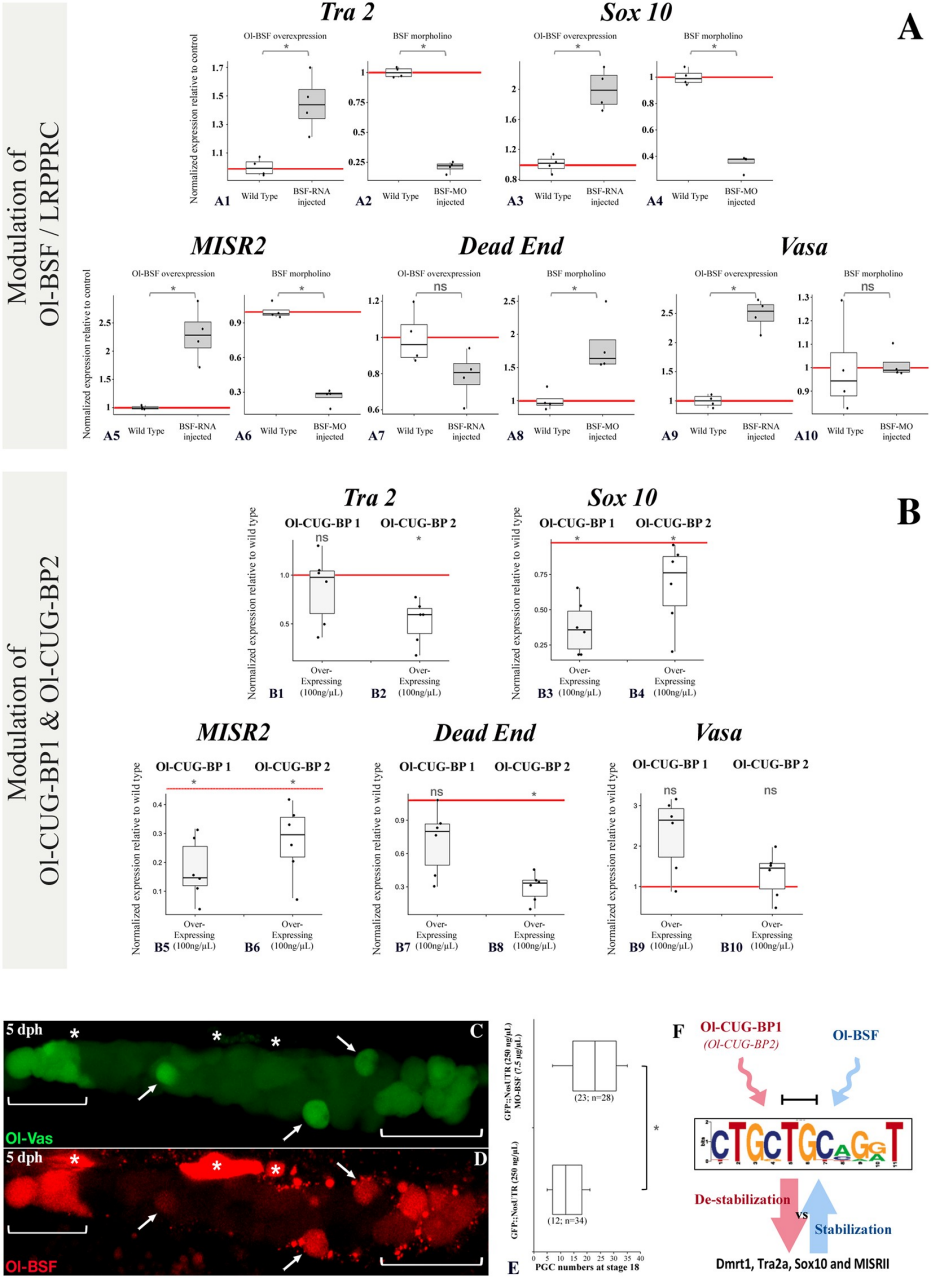
In vivo transcriptional regulation of putative gene candidates harbouring the D3U-box motif in their transcripts after modulation of the Ol-bsf or Ol-cug-bp expressions and correlation between Ol-bsf and Ol-vas expression in germ cells. (A1 to A10) Real-time qPCR determination of expression of putative Ol-BSF-regulated transcripts in embryos at stage 18 after injection of either capped mRNAs with the full Ol-bsf ORF (left odd-numbered panels) or a splice morpholino targeting the exon2-intron2 splice junction (S3 Fig) of the *Ol-bsf* gene (right even-numbered panels) at one cell stage. Ol-bsf overexpression results in an apparent increase of most RNA target candidates, while reduction of the Ol-bsf expression correlates with a reduction of expression. Dataset results of the analysis of 4 batches of eggs injected with either Ol-bsf or Ol-bsf-MO. Statistical significance has been assessed by mean of the Wilcoxon-Mann-Whitney test (*N* = 4). (B1 to B10) Real-time qPCR determination of expression of putative Ol-cug-bp–regulated transcripts in embryos at stage 18 after overexpression of either Ol-cug-bp1 or Ol-cug-bp2. Results are presented as normalized expressions compared to wild type using 3 different housekeeping genes. Dataset results of the analysis of 2 batches of eggs injected with either Ol-cug-bp1 or Ol-cug-bp2. Statistical significance has been assessed by mean of the Wilcoxon-Mann-Whitney test (*N* = 4). (C and D) Germ cell expression of Ol-bsf in comparison to Ol-vas in double transgenic fluorescent reporter lines. After hatching expression levels of Ol-bsf and Ol-vas become more heterogeneous among germ cells (brackets at the tips of the primordial gonad) although tightly correlated between each other within individual germ cells (arrows; ‘*’ = autofluorescent pigment cells). (E) In vivo modulation of PGC number after Ol-bsf morpholino injection. An apparent increase in PGC number is observed after negative regulation of Ol-bsf expression in early embryos (stage 18). Statistical significance has been assessed by means of the Wilcoxon-Mann-Whitney test (*N* = 34 and 28 for wild-type and MO-bsf-injected embryos, respectively). (F) Model for D3U-box–mediated mRNA regulation. Overall and in addition to a cytoplasmic localization of Ol-bsf (Fig 4A), Ol-bsf and O-cug-bps might mutually antagonize toward the access to the D3U-box, resulting in either stabilisation (more Ol-bsf binding) or destabilisation (more Ol-cug-bp binding) of the transcripts harbouring the D3U-box. *p ≤ 0.05; **p ≤ 0.01. Underlying data for (A, B, and E) can be found in S1 Data. D3U-box, *dmrt1* 3′ UTR box; MO, Morpholino; ns, nonsignificant; Ol-BSF, Oryzias latipes Bicoid Stability Factor; Ol-CUG-BP, Oryzias latipes CUG-binding protein; PGC, primordial germ cell; qPCR, quantitative PCR.

For bioimaging analyses of protein localization over time, we used expression reporter lines for vasa [11] and Ol-bsf (see Materials and methods), respectively. During embryonic development, we noted a distinct spatially and temporarily restricted expression pattern (Fig 4). From fertilization up to stages 16/17, Ol-bsf is expressed throughout the embryo (Fig 4A). Of note, cell transfection of a tagged version of OL-bsf and subsequent immunohistochemistry revealed that bsf protein is localized in the cytoplasm (insert in Fig 4A). From stage 25 onwards—when germ cells line up on both sides of the embryo within the lateral plate mesoderm—progressively, Ol-bsf expression becomes restricted to the PGCs (Fig 4B to 4G) where it is co-expressed with Ol-vas, a specific germ cell marker in medaka [60]. During the following developmental stages (stages 33/34), expression heterogeneity for Ol-bsf between germ cells became obvious (Figs 4H to 4J and 5C and 5D). This heterogeneity was particularly apparent between 4 to 10 days post hatching (dph), when Ol-bsf is higher expressed at the tips of the forming gonads (curly brackets in Fig 4K to 4P and square brackets in Fig 5C and 5D). In summary, Ol-bsf has a highly dynamic expression pattern, switching from an early somatic to a progressively restricted germ cell expression. Within the germ cell pool, the levels of expression show a significant heterogeneity.

Furthermore, dmrt1bY expression shows heterogeneity between individual germ cells (Fig 4Q and 4R). Being also higher expressed at the tip of the primordial gonads, the expression levels of Ol-bsf and dmrt1bY show a clear positive correlation (Fig 4Q and 4R).

### Expression of Ol-bsf in the germ-line stem cells of adult gonads

**Fig 6:**
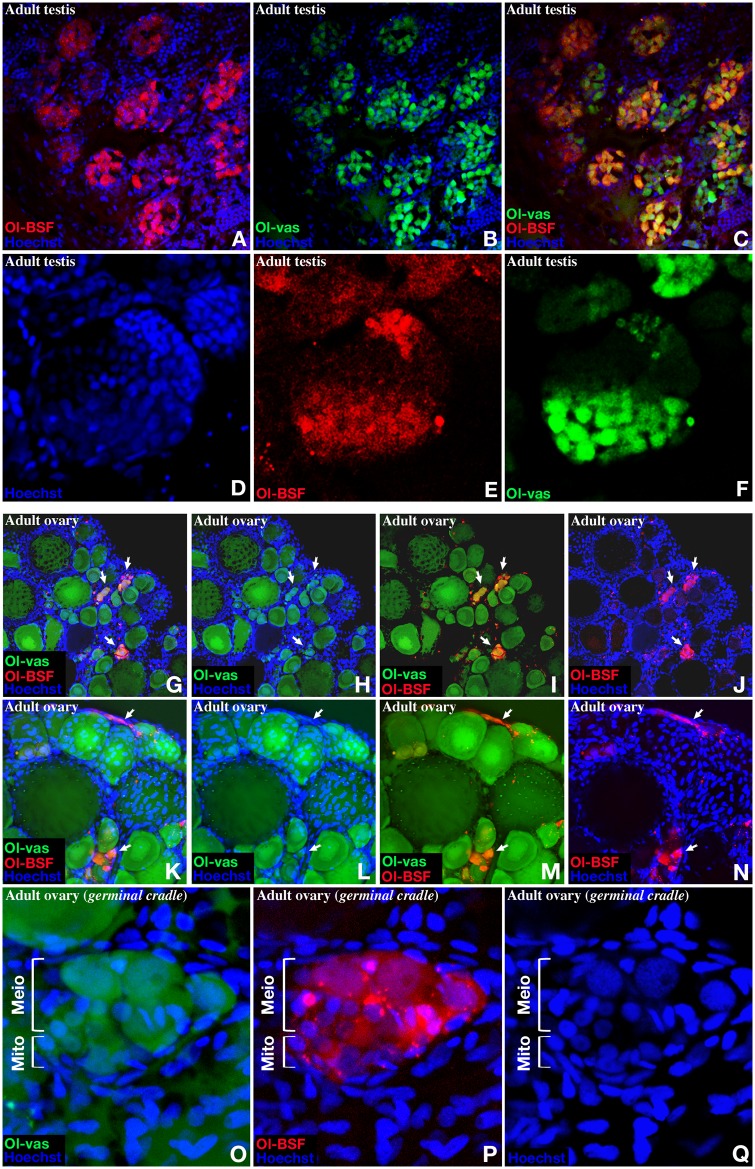
Expression of Ol-bsf in adult gonads. (A to F) In adult testes, Ol-bsf fluorescence (same transgenic lines as in Fig 3) is restricted to the cells located within the lobules and colocalize with a subpopulation of Ol-vas–positive cells. (G to Q) Within the ovary, Ol-bsf fluorescence is restricted to the germinal cradle located in the interwoven threadlike ovarian cords at the periphery of the ovary. The Ol-bsf–positive cells represent the smaller-size subpopulation of Ol-vas fluorescent cells (G to N) as well as early dividing germ cells (O to Q). Arrowheads indicate the germinal cradles (G to N), and brackets indicate early dividing mitotic or meiotic germ cell (O to Q). Ol-BSF, *Oryzias latipes* Bicoid Stability Factor.

Given the high abundance of Ol-bsf transcripts in adult gonads detected by qPCR (S4E and S4F Fig), we next monitored expression of Ol-bsf in fully mature gonads of both sexes at cellular resolution (Fig 6). In adult testes, Ol-bsf fluorescence is restricted to two distinct subpopulations of germ cells, which are also positive for Ol-vas (Fig 6A to 6C). Diagnosed by condensed nuclear morphology and size [62] and localization [39], these first Ol-bsf–positive cells represent the earliest step of germ cell differentiation while another subpopulation of more mature germ cells is also observed (Fig 6C and 6F). In ovaries, Ol-bsf fluorescence is restricted to the germinal cradle [63] located in the interwoven threadlike ovarian cords at the periphery of the ovary (Fig 6G to 6N). These Ol-bsf–positive cells, representing the smaller-size subpopulation of Ol-vas fluorescent cells, are assigned to germline stem cells and early dividing germ cell lineage [63] (Fig 6O to 6Q).

Furthermore, in mice both bsf (lrpprc) and cug-bp1 (celf1) are expressed in the germ cells within the testis cords and germ cells of the ovary (S5A and S5L Fig). Immunohistochemistry revealed that bsf/lrpprc is expressed only in a subpopulation of germ cells in mice (S5M to S5R Fig).

### *Ol-bsf* mutant fish display gonadal phenotypes

**Fig 7:**
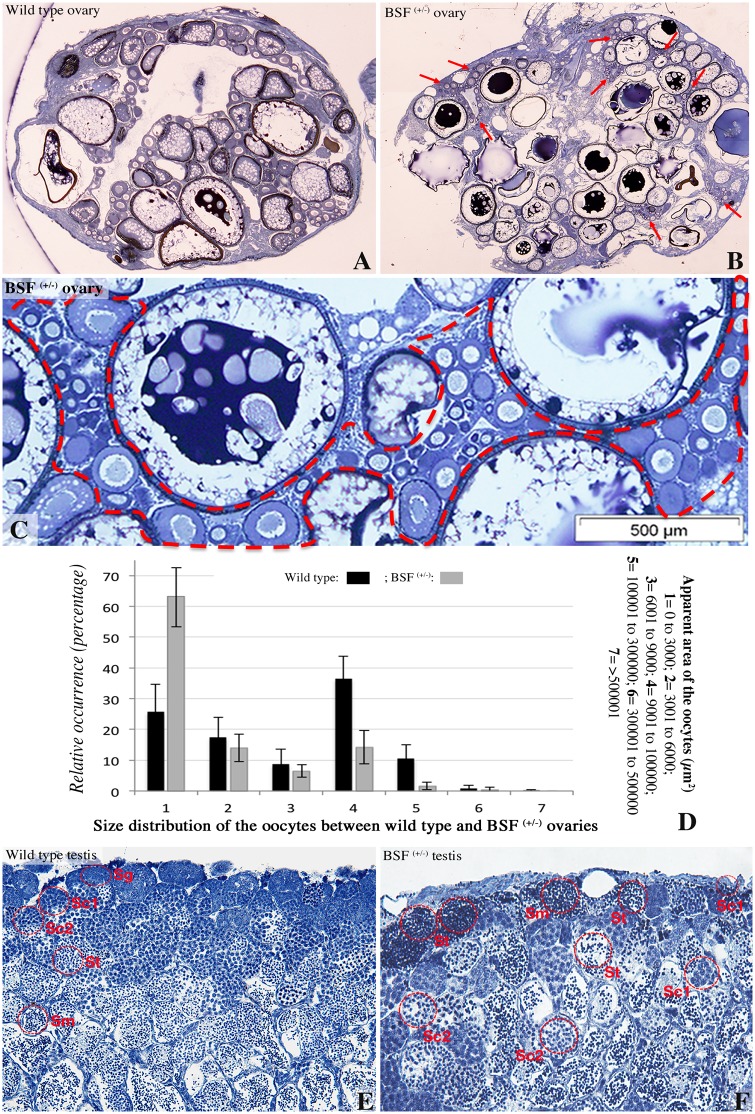
Ovarian and testicular phenotypes of the *Ol-bsf* mutant fish. Heterozygote mutant fish develop normally. (A to D) In-depth morphological inspection of heterozygote mutant ovaries discloses a significant accumulation of small-sized oocytes compared to wild type (dotted lines in [C] and red arrows in [B] and S8 and S9 Figs). (D) Size distribution of the oocytes in 2 wild-type and 2 *Ol-bsf*^(+/−)^ adult ovaries (see also S8 Fig for details). (E and F) Heterozygote mutant testes exhibit a decreased number of spermatogonia with accumulation of type 2 spermatocytes, spermatids, and sperm within the most external layers of the seminiferous epithelium (see also S10 Fig). The different stages of spermatogenesis were determined according to [64]. Each gonad was sectioned through the mid-sagittal plane. Underlying data for (D) can be found in S1 Data. Ol-BSF, *Oryzias latipes* Bicoid Stability Factor; Sc1, type 1 spermatocytes; Sc2, type 2 spermatocytes; Sg, spermatogonia; Sm, sperm; St, spermatids.

To delineate the physiological role of Ol-bsf during gonad formation and maintenance, we generated medaka *Ol-bsf* knockout lines after genome editing using the clustered regularly interspaced short palindromic repeats/CRISPR-associated protein 9 (CRISPR/Cas9) technology (S6 Fig). Homozygous *Ol-bsf* knockout larvae display reduced swimming (S7 Fig) and die within the first 2 weeks after hatching. Heterozygote mutant fish develop normally and produce mature gametes. Histological analysis of gonads of heterozygous mutants of both sexes revealed, however, that ovaries had an accumulation of small-sized oocytes compared to wild type (Fig 7A to 7D, S8 and S9 Figs), whereas testes of heterozygous mutants exhibited reduced amounts of spermatogonial stem cells, with germ cells in advanced stages of spermatogenesis located close to the periphery of the organ (Fig 7E and 7F and S10 Fig). In both sexes, *Ol-bsf* heterozygote mutants present an increase of germ cell committing to gametogenesis. While adult mutant males do not display any observable bias of fertility, adult mutant females have reduced egg production together with lower fertilization rates (S11 Fig).

### Ol-bsf and Ol-cug-bps antagonistically orchestrate expression levels of several germ cell transcripts selectively harbouring the D3U-box

Our matrix scan bioinformatic analysis had revealed the presence of the D3U-box either in 5′, 3′, or coding regions of several germ cell transcripts (S1 Fig). To find out whether Ol-bsf and Ol-cug-bp might regulate stability of these RNAs in a similar way, like for dmrt1bY, during gonadal development the relative abundances of these transcripts harbouring the box motif (tra2, sox10, misr2, dead end, and vasa; see S1 Fig) were first monitored after either overexpression or morpholino knockdown of Ol-bsf in medaka embryos (Fig 5A1 to 5A10). It revealed that for the majority of these transcripts (sox10, misr2, tra2, and vasa), higher Ol-bsf expression correlated with an increased abundance (Fig 5A1 to 5A5 odd numbers and 5A19), while reduced Ol-bsf expression resulted in lower levels of most of these transcripts (Fig 5A2 to 5A6 even numbers). As exceptions, dead end (Fig 5A7 and 5A8) transcript abundance showed either no or an opposite pattern following Ol-bsf expression modulation, while vasa (Fig 5A10) was unaffected in the Ol-bsf-morpholino–treated embryos.

We next checked for the relative abundances of the very same transcripts harbouring the D3U-box (tra2, sox10, misr2, dead end, and vasa) after overexpression of the 2 medaka Ol-cug-bp ohnologs (Ol-cug-bp1 and Ol-cug-bp2, Fig 5B1 to 5B10). With the exception of vasa (Fig 5B9 and 5B10), the majority of the transcripts analysed had lower abundance, whereas medaka Ol-cug-bp ohnologs were overexpressed (Fig 5B1 to 5B8).

In vivo, an apparent correlation between Ol-bsf levels and Ol-vas expression could be visualized in the germ cells of the forming gonadal primordium using fluorescent reporter lines (Fig 5C and 5D). Reduced levels of Ol-bsf expression after morpholino injection led to a significant increase in PGC number at stage 18, (Fig 5E). In line with this observation, microarray data comparing *Ol-bsf*^(−/+)^–deficient testes (displaying reduced levels of Ol-bsf; S12A Fig) to wild type revealed a general up-regulation of genes involved in germ cell proliferation or differentiation. A significant proportion (10.1%) of the down-regulated genes codes for proteins localized in the mitochondria. Finally, gene ontology (GO) term analysis revealed that, in mutant testes partially depleted for the *Ol-bsf* gene, rRNA processing is particularly affected (S12A Fig).

## Discussion

The expression of most genes is dynamically tightly regulated, temporally and spatially. Such regulations occur at multiple steps, including transcription, splicing, mRNA transport, mRNA stability, translation, protein stability, and post-transcriptional modifications [65,66]. While the importance of complex post-transcriptional regulations—like in the case of nanos, oskar, or bicoid, e.g. [61,67–69]—has been mainly identified through genetic approaches for the development of the germline or oocyte, respectively, such approaches have proven to be much less valuable for finding the expected regulatory proteins that bind specifically to these mRNAs. In medaka fish, expression of the master sex determiner dmrt1bY mRNA is very dynamic, occurring first in the PGCs prior to morphological somatic sex differentiation and then quickly switches to an exclusive Sertoli cell localisation [59,70]. Importantly, dmrt1bY is expressed in PGCs of male embryos much before its expression in the pre-Sertoli cells at the sex determination stage [59]. This early PGC expression is necessary for the later onset of dmrt1bY expression in the pre-Sertoli cells at the sex-determination stage of male development [59]. There, the level of dmrt1bY mRNA needs to reach a certain threshold to exert the sex-determining function [21]. This suggested that medaka germ cells exhibit sexually different characters before the formation of the somatic gonadal primordium depending on dynamic and tightly timely regulated mechanisms of post-transcriptional regulations [59,70].

We have identified in medaka a *cis*-regulatory 11-bp motif in the 3′ UTR of dmrt1bY called D3U-box. This motif confers stability to the dmrt1bY mRNA in germ cells of the developing embryonic gonad, whereas in other tissues, the transcript is rapidly degraded [40]. The D3U-box motif was found to be highly conserved in the dmrt1 3′ UTR in the fish lineage (*O*. *latipes*, *O*. *curvinotus*, *Takifugu rubripes*, *Tetraodon nigroviridis*, *Epinephelus coioides*, and *Danio rerio*), as well as in other vertebrates, including *Mus musculus*, *Pan troglodytes*, *Macaca mulatta*, and *Homo sapiens*, and even in the ecdysozoan clade (*Anopheles gambiae* and *Bactocera oleae*).

Both phylogenetic conservation and presence of the D3U-box in several germ cell transcripts implied the existence of similarly conserved *trans*-acting factor(s) involved in the synexpression of those transcripts. To identify such factor(s), we undertook an unbiased approach centred on the D3U-box sequence and based on the evolutionary conservation of the ‘split’ motifs of the D3U-box, implying evolutionary conserved *trans*-acting factors. Further bioinformatics analyses and literature searches revealed that the D3U-box motif is a putative target for 2 RNA-binding proteins, namely cug-bp [45,47] and bsf (also known as lrpprc in mammals [41,42,71]).

EMSAs indicated that Ol-bsf and Ol-cug-bp1, but not Ol-cug-bp2, specifically target and interact with the different parts of the D3U-box, the 3′ and the 5′ parts, respectively. Additionally, our results suggested that the observed regulation of *dmrt1bY* transcript abundance is likely to be the result of a differential binding of the 2 RNA-binding proteins (Ol-bsf and Ol-cug-bp1) with antagonistic properties, *trans*-regulating RNA stability via the D3U-box.

Being—like its mammalian counterparts—ubiquitously expressed, Ol-cug-bps are highly expressed in the gonads of both sexes in medaka (S4C to S4F Fig) and mice ([72] and S5 Fig). Interestingly, Ol-bsf is specifically expressed in the germ cells during medaka early gonadal primordium formation as well as in the adult ovary and is cytoplasmically localized. This expression pattern resembles the subcellular localization of *Drosophila* bsf, which is present in cytoplasmic particles in oocytes and surrounding nurse cells [41] and in the cytoplasms and nuclei in early embryos [42].

In vivo, using fluorescent reporter medaka fish lines, we could show that, besides obvious correlated expression levels between Ol-bsf, dmrt1bY, and Ol-vas in a subpopulation of germ cells of the forming gonadal primordium, medaka Ol-bsf is also preferentially expressed in adult germline stem cells. This restricted and up to now unreported expression pattern might reveal another so far underappreciated role for bsf/lrpprc in germ cell physiology. Similarly, immunohistochemistry localization of mouse bsf/lrpprc revealed its presence in a subpopulation of germ cells in mice (S5 Fig), suggesting an evolutionary conserved function that is not restricted to exclusive regulation of dmrt1.

Reduced expression of bsf/lrpprc in mammalian cell lines [73,74] or flies [75] or because of a missense mutation in human (French Canadian Leigh Syndrome [76]) resulted in decreased levels of mitochondrial mRNAs. This led to respiratory chain dysfunction and increased lactate levels in flies and humans [75,76]. Similarly, in medaka, many transcripts with mitochondrial function displayed decreased steady-state levels when Ol-bsf expression was reduced (S12B Fig). Although knockdown of *bsf* in flies affects climbing ability, fecundity, and life span [75], mutant medaka hatchlings for *Ol-bsf* comparably displayed a significantly reduced swimming ability (S7 Fig), suggesting—with all the necessary notes of caution, such as in *Drosophila*—possible muscle weakness in relation to mitochondrial dysfunction and energy metabolism failure [75].

*Ol-bsf*–heterozygous mutant fish develop normally. Although adult mutant males do not seem to display any observable bias of fertility, adult mutant females have reduced egg production together with significantly lower fertilization rates. In-depth morphological inspection of heterozygote mutant gonads of both sexes revealed that ovaries display an important accumulation of small-sized oocytes compared to wild type. Mutant testes exhibited a discontinuous spermatogenetic flux, likely reflecting uncontrolled spermatogenesis, independent of the seminiferous epithelial cycle. Such a testicular phenotype can be interpreted in the light of dmrt1 loss of expression as a result of low Ol-bsf expression. Similarly, in mice, loss of dmrt1 in germ cells uncouples meiotic initiation from the seminiferous epithelial cycle, resulting in uncontrolled spermatogenesis, too [26]. The mouse celf1 (also known as cugbp1) is predominantly expressed in testis ([72] and S5E and S5F Fig). There, it was demonstrated that celf1 post-transcriptionally represses cyp19a1 (*aromatase*) mRNA translation, by direct binding, to maintain high concentrations of testosterone compatible with spermiogenesis [77]. This situation is reminiscent of the situation we observed in medaka with a strong repression of aromatase expression when both Ol-cug-bps are overexpressed (S12C1 and S12C2 Fig). Additionally, as observed in medaka, mouse celf/cug-bps and bsf/lrpprc are expressed at relevant levels in germ cells, possibly implying a functional conservation across vertebrates.

Whole transcriptome scans using either the medaka or *Drosophila* D3U-box sequences as query resulted in hits enriched for genes specifically expressed in the germ cells, including tra2, sox10, misr2, dead end, and vasa. We provide evidence that Ol- and Ol-cug-bps antagonistically regulate the expression of germ cell transcripts harbouring the D3U-box motif. Certainly most of these regulations occur via an mRNA decay versus stabilization equilibrium after Ol-cug-bps and Ol-bsf targeted the D3U-box motif.

Lastly, to explain the observed differences in the degree of regulation of D3U-box containing transcripts after either Ol-bsf or Ol-cug-bp1/2 modulations, we consider that this follows the degree of conservation of the D3U-box (S1 Fig). It is also intuitive that the location of the D3U-box (5′, 3′ or coding regions) within the transcripts is of relevance (S1 Fig). For efficient regulation, reasonably high (tra2) or moderate (sox10) conservation and location within the UTRs appears to be more effective than a moderately conserved motif located in the coding sequences (vasa and dead end). However, multiple, highly conserved D3U-boxes nested within the coding region seem to be efficient as well (misr2). Dead end transcripts, for which the D3U-box was identified within the 5′ UTR, however, has only strict conservation for the 3′ part (CUGCUG) and is only regulated by Ol-cug-bp1 and Ol-cug-bp2 (Fig 5B7 and 5B8) while it expectedly escapes Ol-bsf regulation (Fig 5A7 and 5A8). Altogether, using complementary approaches, our data suggest that the D3U-box motif is—depending on the cellular context—targeted by 2 antagonizing RNA binding proteins, promoting either RNA stabilization in germ cells or degradation in the soma. This new mechanism of dmrt1 RNA stability appears to also regulate the abundance of other transcripts specifically expressed in PGCs, depending of the preservation of the D3U-box motif.

## Materials and methods

### Bioinformatic analyses

Gene, transcript, and UTR annotation, coordinates, and sequence for human, mouse, medaka, and *Tetraodon* were retrieved from EnsEMBL using the EnsEMBL API (version 54). UTR regions spread across several exons were stitched together per transcript, and the resulting sequence was scanned for the presence of the D3U-box consensus matrix and the other motifs.

### Fish maintenance and breeding

All medaka fish used in this study were taken from closed breeding stocks of the wild-type Carolina Biological Supplies (Carbio) strain or transgenic lines produced on a wild-type Carbio background and were kept under standard conditions [5]. Medaka embryos were staged according to Iwamatsu [78]. The fish used in this study from aquaria housed stocks were kept and sampled in accordance with the applicable EU and national German legislation governing animal experimentation. We hold an authorization (568/300-1870/13) of the Veterinary Office of the District Government of Lower Franconia, Germany, in accordance with the German Animal Protection Law (TierSchG).

### Cell culture, transient cell transfection, and immunohistochemistry

Medaka embryonic stem cells (MES-1) were cultured as described [79]. For transfection, cells were grown to 80% confluence in 6-well plates and transfected with 5 μg expression vector using FuGene (Roche, Germany) reagent as described by the manufacturer. After *pCS2*::*OL-LRPPRC*:*3XFLAG* transfection for 48 hours, cells were fixed in 4% paraformaldehyde (PFA) for 15 minutes, washed with phosphate-buffered saline (PBS) buffer, and then permeabilized with 0.1% Triton X-100 in PBS for 10 minutes. After blocking in 1% Bovine Serum Albumin (BSA) for 20 minutes, cells were incubated overnight at 4°C in blocking buffer (1% BSA) together with the primary antibody (3-times FLAG, monoclonal anti-FLAG M2, category number F1804; Sigma-Aldrich). After extensive washes in PBS, cells were then incubated with Alexa 488 conjugated secondary antibody in 1% BSA for 1 hour. Cell nuclei were stained with Hoechst 33343 (Invitrogen) for 5 minutes (1 μg/mL final concentration) and subsequently mounted using Mowiol 4–88 (Roth). Confocal images were acquired using a Nikon Eclipse C1 laser-scanning microscope (Nikon) and were fitted with a 60× Nikon objective (PL APO, 1.4 NA) and Nikon image software.

### Phylogenetic analysis

The lrpprc phylogenetic tree was built using the online phylogeny.fr automatic pipeline [80]. *lrpprc* sequences were retrieved from public database sequences in the following species and lrpprc homologs were retrieved by tblastn searches on the PhyloFish [81] database (http://phylofish.sigenae.org/) using medaka protein (*O*. *latipes*, XP_011482612.1) as bait. Sequences were aligned with MUSCLE (version 3.8.31) configured with default settings. After alignment, ambiguous regions were removed with Gblocks (version 0.91b) using the following parameters: minimum length of a block after gap cleaning of 10, no gap positions allowed in the final alignment, rejection of all segments with contiguous nonconserved positions bigger than 4, and a minimum number of sequences for a flank position of 85%. The phylogenetic tree was reconstructed using the neighbour joining method implemented in the BioNJ program [82] with *N* = 100 bootstrapping. The resulting phylogenetic tree was exported as a Newick file and edited in Evolview [83]. The public database for lrpprc sequences is as follows: *Cyprinodon variegatus* (XP_015233698.1), *Haplochromis burtoni* (XP_005930623.2), *Fundulus heteroclitus* (XP_012720452.1), *Xiphophorus maculatus* (XP_005804231.1), *Poecilia reticulata* (XP_008426945.1), Lates calcarifer (XP_018552533.1), *Salmo salar* (XP_014035445.1), *Kryptolebias marmoratus* (XP_017287636.1), *Larimichthys crocea* (KKF31900.1), *Pygocentrus nattereri* (XP_017546072.1), *Ictalurus punctatus* (XP_017319936.1), *Austrofundulus limnaeus* (XP_013886428.1), Xenopus tropicalis (NP_001039203.1), *Cynoglossus semilaevis* (XP_008319858.1), *Callorhinchus milii* (XP_007895038.1), *Astyanax mexicanus* (XP_007255829.1), *Chrysemys picta bellii* (XP_005296166.1), *Scleropages formosus* (KPP63655.1), *Bos taurus* (XP_005212770.1), *Cyprinus carpio* (KTG42350.1 and XP_018937975.1), *Equus caballus* (XP_005600080.1), *H*. *sapiens* (BAF82705.1), *Rattus norvegicus* (NP_001008519.1), *M*. *musculus* (AAH59862.1), *Coturnix japonica* (XP_015712995.1), *Serinus canaria* (XP_009094627.1), *Lepisosteus oculatus* (XP_015218688.1), *Amia calva* (AAC_LOC100694568.1), *Esox lucius* (XP_012989242.1), *Latimeria chalumnae* (XP_005999623.1), *Sinocyclocheilus anshuiensis* (XP_016313114.1 and XP_016355337.1), *Sinocyclocheilus rhinocerous* (XP_016417572.1 and XP_016400889.1), *Oreochromis niloticus* (XP_003438484.3), *D*. *rerio* (NP_001136064.1), *Stegastes partitus* (XP_008296301.1), *T*. *rubripes* (XP_011601434.1), *Nothobranchius furzeri* (XP_015800103.1), and *Gallus* (XP_001234903.3). The PhyloFish species are as follows:

- (DAA_LPPRC.1).2_*Anguilla_anguilla*;
- Y_Up_12_k31_Locus_13642_*Umbra_pygmaea*;
- W2_Dp_10_k25_Locus_11398_*Dallia_pectoralis*; S2_Pb_10_k31_Locus_2910_*Pantodon_bulchozi*;
- W_Ha_12_k43_Locus_3235_*Hiodon_alosoides*;
- D2_Om_14_k31_Locus_6093_*Oncorhynchus_mykiss*;
- N_St_1_k25_Locus_84_*Salmo_trutta*;
- O_Ot_10_k25_Locus_4385_*Oncorhynchus_tshawytscha*;
- Q_Sf_1_k25_Locus_1875_*Salvelinus_fontinalis*;
- P_Cl_2_k25_Locus_10853_*Coregonus_lavaretus*;
- I_Tt_3_k25_Locus_2938_*Thymallus_thymallus*;
- E_Aa_2_k25_Locus_4938_*Alosa_alosa*;
- Z_Pa_1_k25_Locus_2290_*Plecoglossus_altivelis*;
- U_Cc_1_k25_Locus_265_*Coregonus_clupeaformis*;
- R_Ok_10_k31_Locus_25108_*Oncorhynchus_keta*;
- G_Ph_4_k37_Locus_379_*Pangasianodon hypophthalmus*;
- K2_Cb_1_k25_Locus_1206_*clarias_batrachus*;
- M_Pf_1_k25_Locus_1736_*Perca_fluviatilis*;
- G2_Ps_1_k65_Locus_195_*Polypterus_senegalensis*;
- K_Gm_1_k43_Locus_6389_*Gadus_morhua*;
- C_Ob_2_k31_Locus_7388_*Osteoglossum bicirrhosum*;
- B2_Gp_10_k25_Locus_1874_*Gnathonemus petersii*;
- V_Sc_10_CL11520Contig1_*Scyliorhinus caniculata*

The celf2 phylogenetic tree was built using the online phylogeny.fr automatic pipeline [80]. *Celf2* sequences were retrieved from Ensembl. Sequences were aligned with MUSCLE (version 3.8.31) configured with default settings. After alignment, ambiguous regions were removed with Gblocks (version 0.91b) using the following parameters: minimum length of a block after gap cleaning of 5, gaps were allowed in the final alignment if they were within an appropriate block, all segments with contiguous nonconserved positions bigger than 8 were rejected, and a minimum number of sequences for a flank position of 55%. The phylogenetic tree was reconstructed using the neighbour joining method implemented in the BioNJ program [82] with *N* = 100 bootstrapping. The resulting phylogenetic tree was exported as a Newick file and edited in Evolview [83]. The genomic context around *celf1* and *celf2* was analysed using the Genomicus website [84]. A few additional genomes were analysed manually by blasting (tblastn) some fish NCBI genomes with the spotted gar Celf protein and by manually extracting the NCBI gene annotation around these corresponding *celf* loci.

### Locomotor activity

Locomotor activity was determined at hatching stage (stage 39, 9 dpf) either under dark conditions or with 10% light (approximately equal to 100 lux) by measuring the total distance swum during a period of 5 minutes (described in [85]) for 12 hatchlings in each condition. Briefly, the larvae were transferred into 12-well plates containing 1 mL Danieau’s solution, and the plate was placed in a Zebrabox equipped with a video camera, infrared light, and filter (ViewPoint Life Sciences, Lyon, France) and the ZebraLab Videotrack software (ViewPoint Life Sciences) for tracking. Following a 5-minute-long habituation period with the same light conditions as for the experimental period, the locomotor activity was recorded. The detection threshold was 11, the inactive/small threshold was 0.5 cm/s, and the small/large threshold was 1.0 cm/s. The total distance swum for each individual is equal to the sum of distances reached during inactivity, small and large movements. *p*-Values were calculated using two-tailed unpaired *t* test with Excel 14.4.8 (Microsoft), and *p* < 0.05 was considered significant. Bars and error bars indicate mean ± standard deviation.

### EMSA

Binding assays were carried out as previously described [38,40]. In detail, 5′-UGGUUCACGU**CUGCUGCAGGU**CUCUGACUCU-3′ for the native D3U-box, 5′-UGGUUCACGU**CUGCUGCAGCU**CUCUGACUCU-3′ for the Olive fruit fly box (Off-box), 5′-UGGUUCACGU**UCUUCAAGACG**CUCUGACUCU-3′ for the D3U-scrambled box (S-D3U-box), and 5′-UGGUUCACGU**UCUUCACAGGU**CUCUGACUCU-3′ for the D3U-minus CUG box (CUGminus-D3U-box) were synthesized and end-labelled. For radioactive labelling, 50 pmol of the duplex 5′ termini were used, together with 50 pmol of gamma-[^32^P]-ATP and 20 units of T4 PNK in 1×-adjusted T4 PNK buffer, and were incubated for 20 minutes at 37 °C. Unincorporated nucleotides were removed through a Sephadex G-50 spin column. For producing Ol-lrpprc, Ol-cug-bp1, and Ol-cug-bp2 proteins, *pCS2*::*OL-LRPPRC*, *pCS2*::*OL-CUG-BP1* or *pCS2*::*OL-CUG-BP2* plasmids were linearized using *KpnI* and then in vitro transcribed using mMessage mMachine kit (Ambion). Finally, Ol-bsf, Ol-cug-bp1, and Ol-cug-bp2 proteins were in vitro translated using Ambion’s Retic Lysate Kit from the previously in vitro–transcribed Ol-lrpprc, Ol-cug-bp1, and Ol-cug-bp2 capped RNAs. DNA binding reaction contained 10 mMTris-HCl (pH 7.9), 100 mM KCl, 10% glycerol, 5 mM MgCl_2_, 1 μg torula rRNA, 0.075% Triton X-100, 1 mM DTT, 1 μg BSA, 0.5 ng radiolabelled probe, and 2 or 4 μL in vitro translation mix in a total volume of 20 μL. The amount of 1/10 volume heparin (50 mg/mL) was added just before loading the binding reaction. For control, reticulocyte lysate alone together with radiolabelled duplex probe was used and did not result in any shift. Binding reactions were performed on ice for 10 minutes, and complexes were resolved on a 5% native acrylamide (37.5:1) gel in 0.5 X TBE and then directly subjected to autoradiography. Of note, due to different exposure times—which can be appreciated through the different intensities of the free probes in the respective figures—autoradiographies should not be compared between each other.

### Establishment of transgenic fluorescent reporter lines and imaging

For a dynamic and in vivo visualization of endogenous Ol-bsf expression, a transgenic fluorescent reporter line was created. The *Ol-bsf* upstream promoter region of the *Ol-bsf* gene (732 bp up to the next upstream gene) was cloned (*BamH1* sites) in front of the mCherry ORF of a meganuclease plasmid (BamH1-BSFp-Fw: AAAGGATCCAGTGTGAGTTCTATCAAGCCTGG; BamH1-BSFp-Rv: AAAGGATCCTTCTGTAGCTGCGTAGAGGAAGATC). For the generation of a stable transgenic line, the meganuclease protocol was used [86]. Briefly, approximately 10 to 15 pg of total vector DNA in a volume of 500 pL injection solution containing *I-SceI* meganuclease was injected into the cytoplasm of 1-cell–staged medaka embryos (Carbio strain). Adult F0 fish were mated to each other, and the offspring were tested for the presence of the transgene by fluorescence check. Siblings from positive F1 fish were raised to adulthood and tested again for fluorescence. Tg[*vasa*:*GFP*] and Tg[*dmrt1bY*:*GFP*] transgenic lines were described earlier [11,29,48,59].

### Visualization of PGCs

For PGC visualization and counting, the GFP-nos1 3′ UTR construct that includes the mmGFP5 ORF cloned upstream of the 3′ UTR of the zebrafish *nanos1* gene [5,87] was injected at 1 cell stage. *N* = 34 and 28 for wild-type and MO-bsf–injected embryos, respectively. For imaging, embryos, hatchlings or tissues were mounted with 1.2% low melting temperature agarose.

### In vivo expression regulation analyses and real-time PCR

For producing Ol-bsf, Ol-cug-bp1, and Ol-cug-bp2 capped mRNAs, *pCS2*::*OL-BSF*, *pCS2*::*OL-CUG-BP1*, or *pCS2*::*OL-CUG-BP2* plasmids were linearized using *KpnI* and then in vitro transcribed using mMessage mMachine kit (Ambion). For overexpression, 1 nanolitre was injected into the cytoplasm of 1-cell–stage medaka embryos. For mRNA stability assays, equimolar amounts of control and D3U-box–containing RNAs were injected.

Total RNAs were extracted from fish tissues or embryos using the TRIZOL reagent (Invitrogen) according to the supplier’s recommendation. After DNase treatment, reverse transcription was performed with 2 μg total RNA using RevertAid First Strand Synthesis kit (Fermentas) and random primers. Real-time quantitative PCR was carried out with SYBR Green reagents, and amplifications were detected with an i-Cycler (Biorad). All results are averages of at least 2 independent reverse transcription reactions. Error bars represent the standard deviation of the mean. Relative expression levels (according to the equation 2–DeltaCT) were calculated after correction of expression of elongation factor 1 alpha (ef1alpha).

### Morpholino injections

For Ol-bsf knockdown experiments, embryos were injected with a splice morpholino: 5′-TTGATGACTGGCCTGCCAACCTGTC-3′ targeting the 3′ splice junction of *Ol-bsf* exon 2 (see S3 Fig). The most efficient dose (4 mg/mL) was experimentally determined and the specificity of the targeting confirmed in control experiments (see S3 Fig).

### Microarray

Total RNAs were extracted from adult medaka gonads using the Tri-reagent (Sigma-Aldrich) according to the supplier’s recommendation. The total RNA yield was estimated using a Nanodrop ND-1000 spectrophotometer (Labtech, Palaiseau, France), and RNA integrity was checked by means of an Agilent Bioanalyzer (Agilent Technologies, Massy, France). Medaka gene expression profiling was conducted using an Agilent 8x60K high-density oligonucleotide microarray (GEO platform GPL24100). Labelling and hybridization steps were performed following the ‘One-Color Microarray-Based Gene Expression Analysis (Low Input Quick Amp labelling)’ Agilent protocol. Briefly, for each sample, 150 ng of total RNA was amplified and labelled using Cy3-CTP. Yield (>825 ng cRNA) and specific activity (>6 pmol of Cy3 per μg of cRNA) of Cy3-cRNA produced were checked with the Nanodrop. The amount of 600 ng of Cy3-cRNA was fragmented and hybridized on a sub-array. Hybridization was carried out for 17 hours at 65 °C in a rotating hybridization oven prior to washing and scanning with an Agilent Scanner (Agilent DNA Microarray Scanner, Agilent Technologies, Massy, France) using the standard parameters for a gene expression 8x60K oligo-array (3 μm and 20 bits). Data were then obtained with the Agilent Feature Extraction software (10.7.3.1) according to the appropriate GE protocol (GE1_107_Sep09). The arrays were normalized (scale normalization) and log-transformed using Genespring Software (version 14.5). A *t* test analysis (*p* < 0.05) was employed to determine the genes that were the most differentially expressed between the 2 conditions. Microarray data sets have been deposited to the GEO-NCBI with the accession number GSE 104726. GO was performed using the panther program (http://geneontology.org/).

### CRISPR/Cas9 genome editing

Identification of CRISPR/Cas9 target sites and design of oligonucleotides of were performed by the use of the ZiFiT software (http://zifit.partners.org/ZiFiT/Disclaimer.aspx). For preparation of sgRNAs, the DR274 plasmid (Addgene number 42250) was first linearized with *Bsa1*, electrophoresed in a 2% agarose gel, and purified. Pairs of complementary oligonucleotides were annealed (40 mM Tris–HCl [pH 8.0], 20 mM MgCl_2_, and 50 mM NaCl buffer) by heating at 95 °C for 2 minutes and then cooled down slowly to 25 °C within 1 hour. The double-stranded oligonucleotides were then ligated into the linearized pDR274 vector. Different sgRNAs were designed to target several sites within the Ol-BSF gene (see S6 Fig) in order to create deletions. After linearization with *Dra1* and *Not1*, respectively, pDR274 and pCS2-nCas9n plasmids were used for generating either sgRNAs or *Cas9* RNAs. The synthesized RNAs were then injected into 1-cell–staged embryos at the following concentrations: 25 ng/μL for each sgRNAs and 100 ng/μL for the *Cas9* mRNA. CRISPR-positive fish were then screened for mutations using PCR primers flanking the site of deletion (see S6 Fig). The inferred mutant protein is presented in S6C Fig. Mutant fish used in this study have been outcrossed for at least 5 generations.

### Histology

Gonads were fixed for 48 hours in Bouin-Holland fluid and then dehydrated serially in aqueous 70% and 95% ethanol, ethanol/butanol (5∶95), and butanol. Tissues were embedded in paraffin, and 5-μm mid-sagittal gonad sections were stained with Regaud’s haematoxylin and haematoxylin–eosin–safran.

## Supporting information

S1 Fig Presence of the D3U-box in different UTRs and coding sequence of gonadal genes. Presence of the D3U-box (matrix-scan) was evaluated within the transcriptome of the medaka fish. D3U-box, *dmrt1* 3′ UTR box.
(DOCX)

S2 Fig Phylogeny and synteny analysis of *Ol-bsf* and *Ol-cug-bp* ohnologs. (A) Circular cladogram representation of the phylogenetic tree of lrpprc proteins in jawed vertebrates (gnatosthomes). This phylogeny shows that *lrpprc* genes were retained as single copies in most jawed vertebrates even following whole genome duplications (red stars), e.g., the teleost-specific duplication or the salmonid–specific duplication. *Lrpprc* is, however, present in duplicated copies in the Cyprininae (tree branches in red). Bootstraps (*N* = 100) values are indicated in each tree node when judged significant (>0.75). Tree branches are depicted in blue for lobefin vertebrates and cartilaginous fish and in black for teleosts with the exception of Cyprininae in red. (B) Gene evolution of *celf2* genes in some teleosts. The phylogeny on the left is a dendogram representation of *celf2* gene phylogeny in teleosts given as an indication as only a few nodes are supported by good bootstraps’ values (*N* = 100, mentioned in each tree nodes when judged significant, i.e., >0.7). The teleost fish whole genomic duplication (3R) is indicated by a red star. The left part of the figure is a representation of the evolution of the genomic context around the *celf2* gene. After the 3R whole genome duplication, *celf2*—which is a single copy gene on the Chr 8 of the spotted gar genome—was duplicated in two 3R ohnologs, *celf2a* and *celf2b*, that were not retained as 2 copies in all teleost fish. The genomic context of the *celf2a* and *celf2b* paralogous regions clearly indicates a partition of the ancestral region found in spotted gar. The *celf2a* gene was retained in all species investigated, but the *celf2b* gene seems to have been lost in Otophysi or at least in *D*. *rerio* (Cypriniformes), *Astyanax mexicanus* (Characiformes), and *Ictalurus punctatus* (Siluriformes). *celf2*, CUGBP Elav-like family member 2; Chr 8, Chromosome 8; Lrpprc, leucine rich pentatricopeptide repeat containing; Ol-BSF, *Oryzias latipes* Bicoid Stability Factor; Ol-CUG-BP, *Oryzias latipes* CUG-binding protein.
(TIF)

S3 Fig Analysis of morpholino efficiency and level of Ol-bsf down-regulation. For in vivo transient down-regulation of Ol-bsf, a splice morpholino was designed to encompass the splice junction between exon 2 and intron 2 of the *Ol-bsf* gene in order to induce aberrant splicing and frame shit of the ORF. To show to what extend the splicing/activity of *Ol-bsf* was impacted, RT-PCR using exons 1, 2, and 3 spanning primers together with cDNAs from different stages of morpholino-injected embryos was achieved. E2, exon 2; i2, intron 2; Ol-BSF, *Oryzias latipes* Bicoid Stability Factor; RT-PCR, Reverse Transcription-Polymerase Chain Reaction.
(TIF)

S4 Fig Real-time PCR quantification of Ol-cug-bp1, Ol-cug-bp2, and Ol-bsf during embryogenesis and in adult tissues. (A and C) During embryonic development, both Ol-cug-bp ohnologs are expressed in a complementary manner. Being likely maternally deposited the expression of Ol-cug-bp1 rapidly decreases after mid-blastula transition (stage 10) to remain virtually off up to hatching stage. On the other hand, low expression of Ol-cug-bp2 is detected until stage 25 and rapidly increases by stage 33. (B and D) In adult tissues, both Ol-cug-bp ohnologs are expressed in brain, muscles, and gonads; ol-cug-bp2 is additionally expressed in eyes and skin. Both ohnologs are higher expressed in male gonads than in female gonads. (E and F) In adult tissues, Ol-bsf is ubiquitously present although higher expression is observed in gonads of both sexes. Underlying data for (A to F) can be found in S1 Data.
(TIF)

S5 Fig Lrrprc and celf1, but not celf2, are expressed in mouse embryonic gonads. (A to H) ISHs on sagittal sections of 14.5 dpc mouse embryos showed expression of lrrprc (A to D) and celf1 (E to H) most likely in germ cells within testis cords (B and F) and germ cells within the ovary (D and H). In contrast, no celf2 expression was detected in developing gonads (I–L). However, celf2 expression was detected in other tissues, such as part of the brain and dorsal root ganglia. Scale bars: 1 mm for A, C, E, G, I, and K; 10 mm for B, D, F, H, J, and L. (M–R) Immunofluorescent detection of LRPPRC (M, N, P, Q) and DDX4/VASA (O, R) in adult mouse testes (M–O) and ovaries (P–R). In adult testes, lrpprc is expressed in one subpopulation of germ cells; compared lrpprc staining on (M) and (N) with vasa staining on (O) where most of the germ cells (except some spermatogonia) remain stained by vasa. According to the position of lrpprc-positive cells (arrowheads in M and N) in the seminiferous tubule (not basal and below round spermatids) and to the fact that lrpprc-positive germ cells are those with the largest nucleus, lrpprc-positive cells seem to be spermatocytes at the pachytene stage. In adult ovaries (P–R), lrpprc is mainly expressed into the oocytes of primordial, primary and young secondary follicles (see arrows on [P] and [Q]). Lrpprc staining disappears from the oocyte of secondary follicles that are clearly stained for vasa in (R) (compared stars in [Q] and [R]). Scale bars: 200 μm for M to R. dph, days post hatching; ISH, in situ hybridisation; LRPPRC, leucine rich pentatricopeptide repeat containing.
(TIF)

S6 Fig Generation *Ol-bsf* knockout medaka fish after genome editing by CRISPR/Cas9 method. (A) Several guide RNA were designed in order to target different locations on the *Ol-bsf* gene (targets 1, 2, and 6). (B) After injection of different combinations of guide RNAs together with the Cas9 mRNA, putative edited fish were subjected to RT-PCR using primer sets flanking the cutting sites. Lines displaying deletions within the *Ol-bsf* gene (red stars) were kept for further investigations. (C) Deletions obtained within the *Ol-bsf* gene (left panel) result in a truncated translated Ol-bsf protein (right panel). CRISPR-Cas9, clustered regularly interspaced short palindromic repeats/CRISPR-associated protein 9; Ol-BSF, *Oryzias latipes* Bicoid Stability Factor; RT-PCR, Reverse Transcription- Polymerase Chain Reaction.
(TIF)

S7 Fig Locomotor activity of *Ol-bsf* mutant fish versus wild type. Locomotor activity (*Ol-bsf* mutants versus wild type) was determined at hatching stage (stage 39, 9 dpf) either under dark conditions (A) or with 10% light (approximately equal to 100 lux [panel B]) by measuring the total distance swum during a period of 5 minutes. (C) The total distance swum for each individual is equal to the sum of distances reached during inactivity, small and large movements. Bars and error bars indicate mean ± standard deviation. *N* = 12 for each condition. Underlying data for (C) can be found in S1 Data. dpf, days post fertilization; Ol-BSF, *Oryzias latipes* Bicoid Stability Factor.
(TIF)

S8 Fig Ovarian phenotypes of the *Ol-bsf* mutant fish. Morphological inspection of heterozygote mutant ovaries discloses a significant accumulation of small sized-oocytes compared to wild type (A) and (A1 to A5 for details and statistical analyses). (A) Overall size distribution of the oocytes in 9 wild-type and 9 Ol-BSF^(+/−)^ adult ovaries. Each gonad (testes or ovaries) was sectioned through the mid-sagittal plan (see also Materials and methods). Underlying data for (A) can be found in S1 Data. Ol-BSF, *Oryzias latipes* Bicoid Stability Factor.
(TIF)

S9 Fig Ovarian phenotypes of the Ol-BSF mutant fish. (A to R) Mid-sagittal sections of the ovaries utilized for counting the oocytes in S8 Fig. Each gonad (testes or ovaries) was sectioned through the mid-sagittal plan (see also Materials and methods). Underlying data for (A) can be found in S1 Data. Ol-BSF, *Oryzias latipes* Bicoid Stability Factor.
(TIF)

S10 Fig Testicular phenotypes of the *Ol-bsf* mutant fish. (A–J compared to K–T) Heterozygote mutant testes (A–J) exhibit a decreased number of spermatogonia with accumulation of type 2 spermatocytes, spermatids, and sperm within the most external layers of the seminiferous epithelium (arrowheads) compared to wild-type testes (K–T). Either 10 different wild-type (A–J) or *Ol-bsf*-deficient (K–T) testes were analysed. Mid-sagittal gonad sections were stained with haematoxylin–eosin–safran. Each gonad (testes or ovaries) was sectioned through the mid-sagittal plan (see also Material and methods). Ol-BSF, *Oryzias latipes* Bicoid Stability Factor.
(TIF)

S11 Fig Fertility test. Egg numbers and fertilization rates were recorded over a period of 9 days for the following crosses: (A) male *Ol-bsf* (−/+) × female *Ol*-bsf (−/+); (B) male *Ol-bsf* (−/+) × female wild type; (C) male wild type × female *Ol-bsf* (−/+). Underlying data for (A to C) can be found in S1 Data. BSF, bicoid stability factor.
(TIF)

S12 Fig Microarray data and mitochondrial gene quantification. (A) Microarray. Adult testes of either *bsf*^*+/−*^ or wild-type animals were subjected to microarray (see Materials and methods). GO term analysis reveals that in mutant testes partially depleted for the *bsf* gene, rRNA processing is particularly affected. Ol-bsf and Ol-cug-bp2 are down- and up-regulated, respectively, in mutant animals compared to wild type. Of note, and in accordance with the literature, a significant proportion (10.1%) of the down-regulated genes code for proteins localized in the mitochondria. Finally, supporting our observations that lowering *ol-bsf* transcription (morpholino injection in Fig 7E) resulted in up-modulation of germ cell number and that mutant gonads presented an increase of germ cells committing to gametogenesis (Fig 6), our microarray analysis reveals a general up-regulation of genes involved in germ cell proliferation or differentiation. (B) RNA levels of different mitochondrial genes (Cox1, Cox2, ND1, ND5, and CytB) were quantified by real-time PCR after BSF-morpholino injections and compared to wild type. Most of the mitochondrial genes are down-regulated when the level of Ol-bsf decreases. (C) Modulation of RNA levels of the cyp19a1 (aromatase) gene after overexpression of Ol-cugbp1 or Ol-cug-bp2. Underlying data for (B and C) can be found in S1 Data. GO, gene ontology.
(TIFF)

S1 Table Gene evolution of *cugbp elav-like family member 2* genes in some teleosts. *celf2*, *cugbp elav-like family member 2*. (DOCX)

S1 Data Underlying data. (XLSX)

## Abbreviations

BAC: bacterial artificial chromosome
BSA: bovine serum albumin
BSF: bicoid stability factor
Carbio: Carolina Biological Supplies
celf2: CUGBP Elav-like family member 2
CRISPR/Cas9: clustered regularly interspaced short palindromic repeats/CRISPR-associated protein 9
D3U-box: dmrt1 3′ UTR box
dph: days post hatching
EMSA: electrophoretic mobility shift assay
GFP: green fluorescent protein
GO: gene ontology
Lrpprc: leucine rich pentatricopeptide repeat containing
MES-1: medaka embryonic stem cells
miR-224: microRNA 224
Ol-BSF: Oryzias latipes Bicoid Stability Factor
Ol-CUG-BP: Oryzias latipes CUG-binding protein
ORF: open reading frame
PARN: poly(a)-specific ribonuclease
PBS: phosphate-buffered saline
PFA: paraformaldehyde
PGC: primordial germ cell
WNT4: Wnt family member 4
