# Supplementary material for: A novel evolutionary conserved mechanism of RNA stability regulates synexpression of primordial germ cell-specific genes prior to the sex-determination stage in medaka
Source: PLoS Biol. 2019 Apr 4;17(4):e3000185. doi: 10.1371/journal.pbio.3000185 (PMC6448818; doi:10.1371/journal.pbio.3000185)
Supplement: S1 Fig — Presence of the D3U-box (matrix-scan) was evaluated within the transcriptome of the medaka fish. D3U-box, dmrt1 3′ UTR box. (DOCX) [file pbio.3000185.s001.docx]

**Consensus fish matrix for the D3U-box motif:**

A | 0 0 0 0 0 0 0 4 0 1 0

C | 3 0 0 4 0 0 4 0 0 0 0

G | 0 0 4 0 0 4 0 0 4 3 0

T | 1 4 0 0 4 0 0 0 0 0 4

*** Oryzias latipes dead end (dnd), mRNA**

ACCESSION NM_001164516

complete cds Length=2148

(coding: 84-1202)

Primers used for real-time PCR: [DeadEnd Fw01: gtccccacagccttctgtaa;

DeadEnd Rv01: gcgtggctaaaaagcatctc]

1 acgagcgcaa caactttctg ctgttgatac caaaacgcca agctggggga aagtataaac

61 tttctagatc gtccaacaag aag**atg**gaca atcagagcaa ggtggtgaac ttggagcggg

121 tccaggcact tcaggcctgg gtcaagtcca ccaataccaa actgacccag gttaacggcc

181 agaggaagta tggagggcca cctgatgtgt gggacggtcc cccacctggg gcgcgctgtg

241 aggtctttat cagccagatc ccacgggatg tctacgagga cctgctcatc cccctcttca

301 gctcggtggg ggcactttgg gaattccggc tcatgatgaa cttcagcggt cagaaccggg

361 gcttcgctta cgccaaatac ggcacggccg ccatcgccaa tgatgccatc cacctccttc

421 acggctaccc gctggggcct ggggctcgcc tcagcgtgcg ctgtagcatc gagaagcgcc

481 agctctgcat ccagaacctg ccagcttcca ccaggcagga ggagctgctg caggtgctgc

541 gtttgctgtc tgcgggtgtg gagagtgtcg ccttgaaggc tggccctggc atagaggggg

601 tgtcagccgt tgttgctttc tcttctcatc atgcagcctc catggccaag aaggctctgg

661 gagaagagtt caagaagcag ttctgcttag acatctctat caagtggctc tctgcagaga

721 agcccaaccc ggacaagccc cctcctcaga gagctcctaa aggcctgctg ccgtcccccc

781 tgaagcacct aggccaaacc tcccctcggc tgcccccacg attggcttct cccgcagtcc

841 ccacagcctt ctgtaaagct gtgggtgggc cccccacaca gcatgatact catgttaaag

901 gcacctctcc tccccccagg ggacaagtca tgttttctgt gtccccggtg ctgttgctgc

961 ggaagctgag tgaggcgagt gggtgggggg atccacacta tgagatgctt tttagccacg

1021 ccggcccaga tggatttctg tacttcacct acaaggtgca cgtccccgga gcccccacca

1081 ccttcagggg cttcgtcatg attttaccag gacactgcac gtccaccatg ctggaggagg

1141 ccaggagggc tgctgcccag caggtcctgc agaagttgtg cagcagcggt ttgagcgcc**t**

1201 **ga**acatttta gtgcctgtgt gtttttgtcc tgcatgtttt agggtgttga gttattgcag

1261 tgtgtttgca tgcttttcta ctgttcagtg ttcctcgttt gaagtaaatg agatccaaaa

1321 gaaacgccgt cctctttgtg tagccgcacc accacagctc acagtcctcc tgtagacctt

1381 cagaagaagc ttgtcatcac acatccaaaa acaaaataca catgtaatag attacgagtt

1441 taatgcacag atattattct tttcaaagca agtggtgcta atgcaacagc agacagggcc

1501 agaggccaga tgttacagtt atcaaccctg atggagaaga ttccactgca gtaaagagcc

1561 tgagcagcaa caaacataac cagcattgga ccagaccagc accagaactg ccatagtacc

1621 tgcagcagta aacatgaagg aaggaacctc catttctggt ccatcagtcc ttttttcatt

1681 agaagcttca gttctggttg cagattcagg aagaacacag actctttgga cacgagagct

1741 ttaatgaaca tgtgcagcaa actcttggac caggcaggta aacatctgcc ttgactgcag

1801 gagcagcctg aactcctttc taggccacat cagaaccaca gagctccaag gacaactggg

1861 ggccaggggg atgctgtaca aaactggagt tgggcagcag ggaggagtta ggcttcagac

1921 gtcctcctct tgatctcagc gatgagatcg gctctggaga tgtccacctg cggacagatg

1981 gcagaaatga gctctgttca ttagtttgtg gctcagagct gagcagctaa ggcatcatca

2041 gctgaggtgt aagacgatgt tccaccttga tgactgcaag ggtcacatct gcccctacag

2101 cacagatgga ggacacacca gacccataaa aattaaatct ctgctttc

Matrix scan:

| **strand** | **start** | **end** | **sequence** | **weight** |
| --- | --- | --- | --- | --- |
| D | -2131 | -2121 | CTGCTGTTGAT | 6.7 |
| D | -1624 | -1614 | CTGCTGCAGGT | 11.3 |
| D | -1195 | -1185 | TTGCTGCGGAA | 6.0 |

*** Oryzias latipes transformer 2a (tra2a), mRNA**

ACCESSION AB159270

cds Length=2811

(coding: 1733-2293)

Primers used for real-time PCR: [Tra2a Fw01: tctgggtgtgtttggtctga;

Tra2a Rv01: tccattagcttgctccttgg]

1 gagacagcaa ggttccctct ggggttttcc ctttttgttc tgctggttgt tccatcatgt

61 ggttttcctg ccaaaggctc caaatccaca gatcatggca gtggtgacca aaaagcagca 121 actaactttg cttcccacag aggagctcag atgagccgct ctgctcctgt gcattacagc 181 tctcattcaa gcggcgtctc tcagccagtt ccagtgtttg tgcagaatcc tggtgtccct 241 cagtttgttc ccatggttcc atcaggcagc agcttgagct ctgctgtggc aggataccct 301 gtggctgttc agacaggatc tactgttcct gtgttttccg gtgttcctgc agcaggagtc 361 tctgaggcag ttcaggtccc tgtgcaggct cctggtttct cccagtttgt tcagacgggc 421 ccagcaatgg ttggctctgc ttcccctgtg tttgtgctgc atgaaggagc tggttccaca 481 cagccaggac ctgctcaagc tgctctgcca gaaacccagt gggctgttgc tcctccatct 541 ttctctgagc aagcagcagc tgatgcccag gctgtgggct ccagtgactt cctgccacca 601 gtccctgccc ctccagctgg ccctgtcctc cagtctggag agacttccaa tattgtgaag 661 gaagctgaac ttggcaacta ccagcagcag acggaagagt ttggttaccc ggctgaggcg 721 gcgcagcctg gagctgcttt caccagcgtg cttgttccag gccaaggact tggtggcttc 781 tggggttctc cttatcctgg ctttgactac aggctcctgt atggtctgta ccctcctggc 841 acctacacta ccttcagcca gaaccatgag aagggcaaag actactacca atccatccac 901 tacttgaagg agcatgtttc tgaagaccaa ggtcctcaac agcagcagct tcagcagaag 961 gtcttccatg gtcaacccca gagatcttga ggctcatcaa cgtggaggat atcatgagca 1021 cattcagcct catttagaac ttggatgctt aagtttgacc taataaaaac agatctggaa 1081 aaaaaaatcg gtgtgcgcag cgcttagtta aatttagtga tctcgcgtgt tacgctatca 1141 ttcctgttaa tccttatcaa atttcatagt ttcaaaatga gtgacaccga aaaagaattc 1201 aagaaggagg ttgaggttgg aagaatgcaa aaggctcaag atgtgaagtt ttaaatgttt 1261 gaagctgatg gtgttgatgc atgctaatgt attggagact gaacagaagc gatcaagatc 1321 tgaccccact tctagtgttg ttattggcga tgggatggta aaaatgggga agaggaagag 1381 gaaaaaagaa gtcagaaatt caaacggaag aaagaagaaa tgaagggttg ttatgaagta 1441 atgaagttaa gatcagaagt atacctggga ttcgagaggt cactcgagga gtgcatcccc 1501 tcggggttct gctaaatcta ctagccactc accagcgcgt tcaaaagatg gctcccacca 1561 ttcgaggtcc agatctcgtt ctcgttcaag atcaaaatct aggtctcggt ctcatcgcag 1621 ttcgcgcagg cgctactccc gttctcgttc ccattcccgt cgccggcgtt ccagaagccg 1681 gtctcgtagc tcagactacc gccggcgaca taggagccat agccgctctc cg**atg**tcaaa 1741 tcgccgcaga catattggca acagggggaa cccggaccct agctgctgtc tgggtgtgtt 1801 tggtctgagc ctgtacacca cagagagaga cctgcgcgaa gttttctcca agtacggccc 1861 actgagcgaa gtgaacatag tttatgacca acagtctcgt cgctcgaggg ggtttgcctt 1921 cgtctatttt gaaaattctg aggattccaa ggaggccaag gagcaagcta atggaatgga 1981 acttgatggg cgcaggatcc gggtggattt ttccattacc aaaagagccc acacgccaac 2041 ccctggaatc tacatgggac gtcccacgta tggcggaggc ggtggaggtg gtggtggttc 2101 tcgtcgtggt tcaagagatt atgacagagg gtatgaccgg ggctacgaca gaggttatga 2161 tagagggtat gatcgtgact atgatcgata tgatgatcgg gagtaccgat catacaggcg 2221 cagatctccg tctccatact acagcagggg ataccgatcc agatctcgat cctattcacc 2281 gcgtcactac **tga**acctcag tggccatcgg tgttacttct caatgctttt tttccttttt 2341 ttcagtgttt tgaccagatt ttacatggaa agtcattttg gacatgttgg tatataaaac 2401 atgcgaatgt tttcattaaa atgttacaca tagtcgctca atgtcacaaa tttattgtgg 2461 gaaaacagtg gatcatgtga gaaccggtca tggtgtggat caggtgtgcc ccttatctcc 2521 taaataagtc tttttttctt aaagtggata taagacagat atttgtcagg gaacctgtta 2581 ccatgtgtaa aataaattgc tgaatttgtt aaacattcgc tctaccgatt ttctttttat 2641 ctcacgcatt tttgtcactt gttctttttt ttcctttttt tttttacgat aagtgtcatg 2701 gtcctagttt gtcatatttt agatgtttat gttttatttt ttctttgttt ttgtacacaa 2761 gtaaataaag tcaatagctg tcatcctgtt aaaaaaaaaa aaaaaaaaaa a

Matrix scan:

| **strand** | **start** | **end** | **sequence** | **weight** |
| --- | --- | --- | --- | --- |
| D | -2465 | -2455 | CTGCAGCAGGA | 6.9 |

*** Oryzias latipes Mullerian inhibiting substance type II receptor (misrii), mRNA.**

ACCESSION: NM_001104896

complete cds Length=1687 bp

Coding (15-1502)

Primers used for real-time PCR: [MIS2Rec Fw01: tgtcctggtgagagcagatg

MIS2Rec Rv01: gcaggaaggagctgttgttc]

1 atgagcgctc ttag**atg**ggg aaaatgctgc tgtggattct ggctttatat tgccttaaat

61 atgtgtttcc tcagtccttg cttccaaaga gaacgtgcgt ttttcaagta agccacaaaa

121 acaacggagg acactcatct gctggtaatg tgagtgggtc cctgcagatc tgtgagaaca

181 cccgctgctg catggctatc tatcggatga taaacggcca accacaggtg gacactctcg

241 cttgcggtaa agtggaaacc ttatgtccag atgcaacctg caaggtacaa ccacgccttc

301 aaaatcgctt cataggatgt gtgtgtggaa cagatctctg caacagtaac ttcacgtgga

361 ctccagattc agagcagcat tcactcactg actcctactc tgcagctgac atctcgaaga

421 ttgttgtgtt ggtgatgttt ccaatactcg cctttgcact cgctgctaag tggatttatc

481 tctacaaaaa gaaagagaaa aatctgctgt ctgttcttcc taatgacaac atcctgcaac

541 catgctcttg tcaaacaaag tcttcagaga gttacattac gaacatcgaa ctgcagcagg

601 ttgtgggaca aggacacttt gccacagttt ttaaagggaa ctacacggaa actgcagtgg

661 ctgtgaaagt gtatcctgca gggtggaaag ataaattcac aacagaaaag gacatttatg

721 aattaccact gatgaggcat gctgggattg ctcacttcct gggtgcgggg aggaatccaa

781 gagacagaag ctggtttgtt gtggtcgagt ttgcagaaca tggttctctt cactcctttc

841 tgtgtgaaaa caccctcagc tggatgcagg cacagaacat gtgtctgtcc ttgtcccaag

901 gactttccta tctccactct gacctccaca gccaggatgt gcacaaacct gctgtggccc

961 atagagacct gagcagcttc aatgtcctgg tgagagcaga tggtagctgc gctctctgtg

1021 actttggatg ctccactatt ctgcgttctt gctcagggcc atgggtcatg agacaagcca

1081 caaacacaaa gggttatgcc cagcttggta ctccccacta catgtctcct gagatcctgg

1141 agggctccgt aaacctgaac aacagctcct tcctgctgca ggccgacatc tacgccttga

1201 gtttgatatt gtgggaaata tggatgcgct gctctgattt atcaaagggt ggcaccgttc

1261 cacagcatct tttgccgtat gaactggagc tggaaggcaa cgtgacgctg gagagactca

1321 tcctctatgt gtgtgaaatg gacaggagac cttcaatccc tgaaccctgg gagctgctgc

1381 cacagggatc tgcaatgaag gaactcctga cggaatgttg ggaccgggac acagatgccc

1441 gtttaacagc acactgtgtt gtggacagat tagtgtctct ccaggctgat ttatcacca**t**

1501 **ga**ttattatt attattatgc acatagtttc agttgtaaaa cgcagctgta cactctttgt

1561 tttcactgtt tttttaaatt caagtcaggt agttaaatgt tgaagaactg cacataaagt

1621 atagtgacat attcagatag aggctactca aaatatacat tttcctaggt ggtaaaaaaa

1681 aaaaaaa

Matrix scan:

| **strand** | **start** | **end** | **sequence** | **weight** |
| --- | --- | --- | --- | --- |
| D | -1097 | -1087 | CTGCAGCAGGT | 8.6 |
| D | -515 | -505 | CTGCTGCAGGC | 8.1 |

*** Oryzias latipes DEAD-box helicase 4 (ddx4, vasa), mRNA.**

ACCESSION NM_001104676

complete cds Length= 2187 bp

Coding (94-1947)

1 tcagtttgaa gctaacagca gcacgagaag ccacacgtgt ggaaaggccg cgaggacaag

61 aaaagttctt aaatagttga tcagacgatt aaa**atg**gacg actgggagga agaggaaacc 121 gcaccgtcct tcgcgcccgt cagcagcacg gatgcagctc cgcagagaag ctcctggaac 181 ggaggcgcca gggactcagg aaatgacgga gacagctgga accgctccaa ccgagggcga 241 ggaggttcgg ccgggagagg aggcagagga gggcgcggca gaggcttcgg acggtctgac 301 caggacgagc tcaatggagg cggcggagac tctgaaaacg ggttcagagg aagaggccga 361 ggaggaagag gaggcttcag atcaggtggg ggggagcgag gaagaggagg aggttatcga 421 ggacgggatg aagacgtctt tgctgcagga gacggcagag gggccgagaa cagcgatgca 481 gctgacccag aacggcccaa agtgacctac atccccccga gcctcccgga ggacgaggac 541 tccatcttct cccactacaa gatgggcatc aacttcgaca agtacgacga catcctggtg 601 gacgtcagcg ggaccaacct gcccgccgcc atcatgacct ttgaggaggc caagctgtgc 661 gagtcgttgg agaacaacat cagcaggtcc ggatacgtca agccgacgcc cgtccagaag 721 tacggcctcc ccatcatctc cgccggcaga gacctgatgg cgtgcgctca gaccggctcc 781 ggaaaaactg cggcgttcct gctgcccatc ctgcagcagc tgatggcaga cggcgtggcg 841 gccagccgct tcagcgagat ccaggagcca gaggccgtga ttgtggctcc aaccagggag 901 ctcatcaacc agatttacca ggaggcccgg aagttctcct ttgggacctg cgtgcggccc 961 gtggtggtct acggaggcgt gaacaccggc taccagatga gggagatcga gaagggctgc 1021 aacgtgctgt gcgggactcc gggacgcctg ctggacatga tcggcagagg aaaggtgggc 1081 ctgagtaaag ttcgccacct ggtcctggac gaagccgacc gcatgttgga catgggcttc 1141 gagccggaca tgcgccggct ggtgggctcc ccgggtatgc cgtccaaaga ggagcggcag 1201 acgctgatgt tcagcgccac cttccccgag gacatccaaa ggttggcggc cgacttcctg 1261 aaggtggact acctgtttgt ggccgtgggg gtggtgggcg gagcctgcac cgacgtggag 1321 cagaccttcc tacaggtcac caaattcaac aagcgggagc agctcctgga cctcctcagg 1381 accatcgggt ctgagcggac catggtgttc gtggagacca agaggcaggc cgacttcatc 1441 gccgccttcc tgtgccagga gaaggttccg accaccagca tccacggcga cagagagcag 1501 cgcgagcggg agaaggcgct ggcggatttc cgctcaggca agtgtcccgt cctggtggcc 1561 acctcggtgg cgtcccgcgg cctggacatc cctgacgtcc agcacgtggt gaactttgac 1621 ctgcccaaca ccattgacga ctacgtccac cgcatcggga gaacgggccg ctgcggcaac 1681 accggcaggg ccgtgtcctt ctacgacccc gacgtggaca gtcagctggc ccgctcgctg 1741 gtcggcatcc tggccaaggc tcagcaggag gtgccgtcat ggctggagga gtcggcgttc 1801 ggcgctcacg gttccgccgc cttcaaccct tcgggaagga cgtttgcttc cacagactcc 1861 aggaagggtg gttctttcca ggacagcagt gtgaagacgc agcctgcagc gccccctgct 1921 gctgctgatg aagatgactg ggag**tga**agg ccccgccgcg tcagcttcct cctccactga 1981 atgccgaagc gttccgtttg ttttttttta aggcatttaa acttgtgtta aagtagaaac 2041 tgggaggatg agtgtccgac cgcccacgtt caagtccgga agaagaggat cagaggagcg 2101 ttctcctgac agaatgacta tagagaaaag ttggtattgt tgtatccttc agcggttttc 2161 tcctgataat taaaagtttt caacaaa

Matrix scan:

| **strand** | **start** | **end** | **sequence** | **weight** |
| --- | --- | --- | --- | --- |
| D | -1748 | -1738 | TTGCTGCAGGA | 8.6 |
| D | -1140 | -1130 | CTGCTGGACAT | 6.0 |
| D | -861 | -851 | TTCCTACAGGT | 5.4 |
| D | -272 | -262 | CTGCTGCTGCT | 9.9 |
| D | -269 | -259 | CTGCTGCTGAT | 11.1 |
| D | -266 | -256 | CTGCTGATGAA | 5.4 |
| D | -263 | -253 | CTGATGAAGAT | 5.6 |

*** Oryzias latipes SRY-box 10 (sox10), transcript variant X1, mRNA.**

ACCESSION XM_011478420

complete cds Length= 3539 bp

Coding (445-1899)

1 ttaagccgtg ctgtaggcac acagagcagg cattcattct cgggcggcgc aagcgagggg 61 gctggggagt gagcgtcact ctggaaactc ggggacccgc ggattctcga cctctttcct 121 cactgctgaa gtttttgtgg atttccaatt cgaagagggg gagagaaggt gggacagacg 181 tgaggctggg tgagctttgc gcagacgccc gtgtgtcaga aggtaaactg gggcgcactg 241 agctgcctat gagaccatcg acacggatgg aaagaacttt aatgtggaat cctcacagag 301 ttttgcagcg gaggctgaga gtagagacag actggcagtt tatattataa tttagaggag 361 ttcacagaga cagagcagtc agcagagtcc atcaagcaga gctgagaatt cacaaaaagg 421 ctttaggttt tcctcttgaa gaag**atg**tcc agggaggagc agagcctctc agaggtcgag 481 ctcagccccg gcatgtcgga tgacagctgc tcccagtcac ctggtcattc cctgggggcg 541 gcaggcgcgg gagagtcccc cctccacggg cagcagcatc cgcagatgga cggcgagtcc 601 gcgggctgtt cttccgccaa atccgacgac gaggacgagc gcttcccgga cgggatccga 661 gaagcggtga gccaggtgct gaaatgctat gactggacgc tcgtgcccat gcccgtgcgc 721 gtcaacgcgg gaagcaagaa caagccccac gtgaagaggc cgatgaacgc cttcatggtg 781 tgggcgcagg cggcgcgcag gaaactggcg gaccagcacc cgcacctgca caacgccgag 841 ctcagcaaga cgctggggaa gctgtggagg cttcttaacg agagtgacaa gcgacccttc 901 attgaggagg cagagaggct gcggaagcaa cacaagaaag attatccgga ctacaaatac 961 cagccgcggc gcaggaagaa cggaaagctt ggcacgggat ctggaagtga ggctgacgga 1021 caactggagg gtgaggtcac ccactacaag ggcctccagc tggaagtggc tcatggtggg 1081 ggggccgagt cccttctggc agatgggcac caccctcatg ctgcaggaca gagccacagc 1141 cctcccacgc cccctaccac acccaagacc gaagctctgg ccggaaaagc aggggatggg 1201 aagagggatg gaggggggaa cgggagctca cgcgcctcca tgggggctga aggaggctct 1261 ggtgcttctg ggtccgggaa acctcacatc gactttgata acgtggacat tggagagatg 1321 agccatgaag tgatgacaaa catggagcct tttgatgtca acgagtttga ccagtacctg 1381 ccccctaacg gccatcctgc agttgggcag agcagtgggg ccggaggagc tgccgctgct 1441 gctccgagct cgccatacgg ctatggcatc tcgtcagctt tggcggcggc cagcggacac 1501 tcggcggcct ggctctccaa gcagcagcag catcacagct cccccctggg ggacgcctct 1561 aaggtcaaga gcgaggctgt gacctctggg agtcactttg cagaggcggc ctctgcaggc 1621 acgcatgtta cctacacgcc actcagtctt cctcactaca gctccgcctt cccctcattg 1681 gcctcaagag ctcagttcgc agattatgcc gatcaccagg tctcagggtc ctactacgcc 1741 cactccagtc aggctccggg gttgtactct gcattctcgt acatggggcc ttcccagaga 1801 cccctgtaca ccgccatcac tgaccccgcc agtgtgaccc aaccacacag ccccacccat 1861 tgggagcagc cggtctacac aactctgtcg cggccatgac agatgacaga accaggaggc 1921 gcctgtgccc gcccagcccc aacatcagac cacagaagca gaggcgacag caacagtgga 1981 gctgttggaa gttg**tga**tga aagtgctctg gtctgggggg cttgcgcagg ccgggagggc 2041 ataaggggtg aaaccttgtt ccaatcctgc ccgcgtgtag gagaaaacga tgtaagaaag 2101 cggagggtgg tgtggcagca ctcagtgtgg ttgtgaggga aactggagac ccttcagaaa 2161 ttgcagcagc tgtgccagtg agcgctcagg taggctctca cctgttccaa acttcacggg 2221 tatcaggagg cacactggag tctgcaaaca tcgcccccaa acaaaaaaca aaaaaagaga 2281 aaagcacaac tttccttcct gaaatgaaac caggaaagaa tcattacaca ataacacatt 2341 gccaatcttt tgttgttcat gacataaaat taaagattat aactcaaatg gatgaataaa 2401 tcctgaaatg gttgttttgg caagaatttg tccactttgt tctcagaaaa ccacagttac 2461 ctaaactcaa acagatctgt taaaaaggaa aaaaaatcaa aatgtatcaa tattttaagt 2521 aacaaatgtg ccttctgtag aaatccagac aaactaatga aactatgaca aaataaccaa 2581 agcattgaga ccaaaacatg ttttactgtt ataggataga aacagatttt taaagatgtt 2641 tctcccaaca tgacactcaa agctacctgg tagttcgtag agatgttttt tggattagcc 2701 aaaacatttt cagtaaaagt tattcttctt ttatcagaaa gtttttactt tatcttatct 2761 gaagtttctt ttccttcttt tagtgttttc taataaaatc ttcagacttt tatgtactta 2821 tgacttgaga aaactttatt ttgtactcac aaaaaacagt gaagtttgac taataatatt 2881 tgtattttaa gatttgttaa agaaaaactg ggatctcaca gattactatt aaaacatttt 2941 gccattaact tcacttattg cttcaccact gtctaatttt ttactttttt tgggtaaaat 3001 gttagcattt tgctaatttt aagattgatt cattaaaaaa aacggatttc atacttgtat 3061 gctgaaggag atgccaatat ttttgaatga cttgccacac gtgaagataa tttcagattg 3121 cattaacgaa gtgccttgtt cttgacaatt tacttacatg taattatatt tcattctatt 3181 gtgatgaaaa tccttcaaaa tctacattaa atattattct atgccatatt taaactgctc 3241 gctatgtaaa agtataacaa tgctttgttt tctgtcatgc taagactgga ctgtaacttt 3301 aaattgaaat gtttcttttt gttgctctga gctttttcat ttcctattta taattttttt 3361 tgcatctatt tgtgtaaaaa gcattttttt tctttctctt tgatctaagc tgccatttat 3421 tttaatagga acagtggatt tcagtcacag agtctgtaaa taagagcagg agcaaaagcc 3481 tgaacatgtg acgatgactc tcacttgaaa taaagttctt ctgttttttt gcctgttca

Matrix scan:

| **strand** | **start** | **end** | **sequence** | **weight** |
| --- | --- | --- | --- | --- |
| D | -3417 | -3407 | CTGCTGAAGTT | 5.8 |
| D | -2422 | -2412 | ATGCTGCAGGA | 7.0 |
| D | -2110 | -2100 | CTGCCGCTGCT | 6.4 |
| D | -2107 | -2097 | CCGCTGCTGCT | 6.4 |
| D | -1379 | -1369 | TTGCAGCAGCT | 5.1 |
