# Supplementary figures and images for: A novel evolutionary conserved mechanism of RNA stability regulates synexpression of primordial germ cell-specific genes prior to the sex-determination stage in medaka
Source: PLoS Biol. 2019 Apr 4;17(4):e3000185. doi: 10.1371/journal.pbio.3000185 (PMC6448818; doi:10.1371/journal.pbio.3000185)

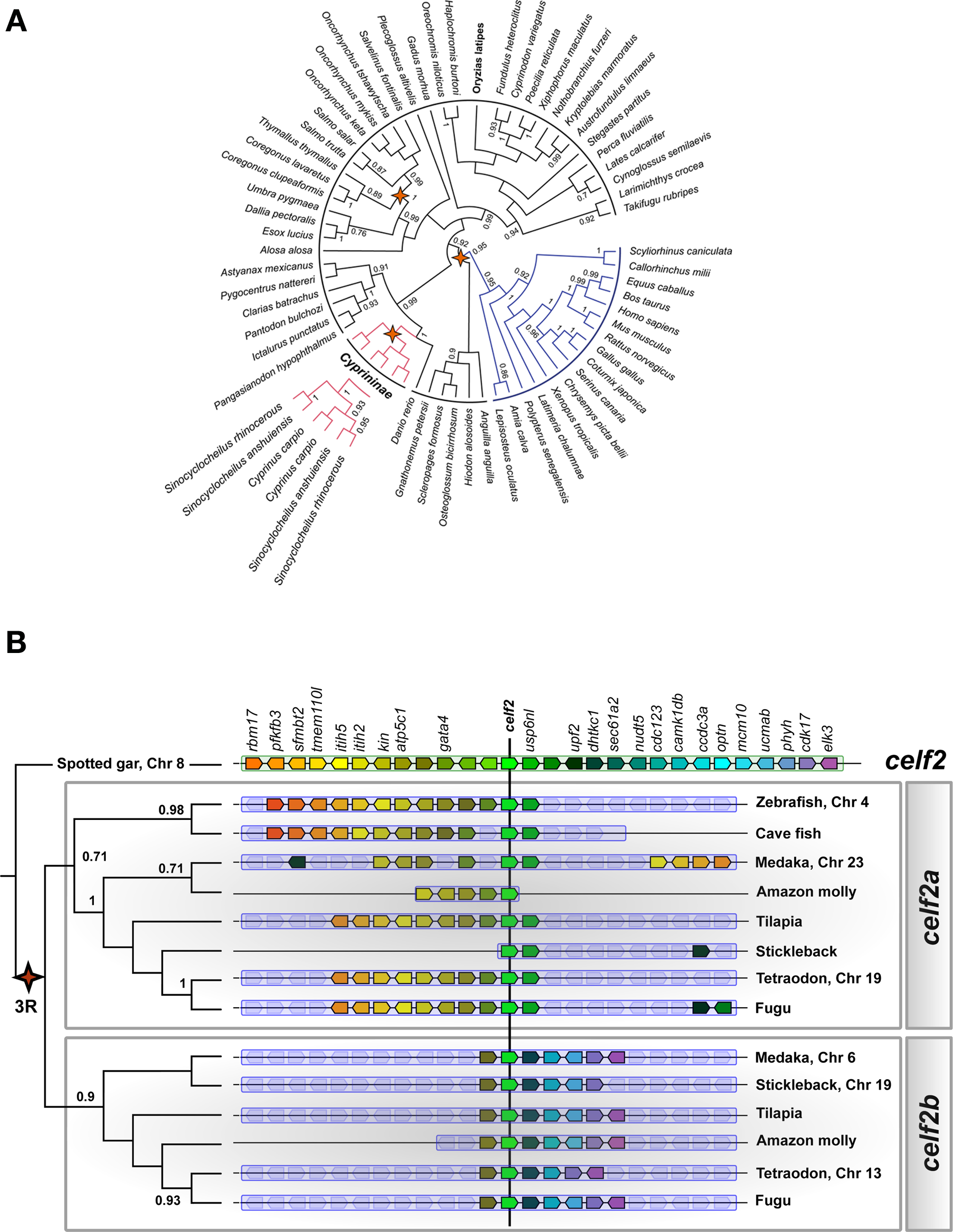

Supplement: S2 Fig — (A) Circular cladogram representation of the phylogenetic tree of lrpprc proteins in jawed vertebrates (gnatosthomes). This phylogeny shows that lrpprc genes were retained as single copies in most jawed vertebrates even following whole genome duplications (red stars), e.g., the teleost-specific duplication or the salmonid–specific duplication. Lrpprc is, however, present in duplicated copies in the Cyprininae (tree branches in red). Bootstraps (N = 100) values are indicated in each tree node when judged significant (>0.75). Tree branches are depicted in blue for lobefin vertebrates and cartilaginous fish and in black for teleosts with the exception of Cyprininae in red. (B) Gene evolution of celf2 genes in some teleosts. The phylogeny on the left is a dendogram representation of celf2 gene phylogeny in teleosts given as an indication as only a few nodes are supported by good bootstraps’ values (N = 100, mentioned in each tree nodes when judged significant, i.e., >0.7). The teleost fish whole genomic duplication (3R) is indicated by a red star. The left part of the figure is a representation of the evolution of the genomic context around the celf2 gene. After the 3R whole genome duplication, celf2—which is a single copy gene on the Chr 8 of the spotted gar genome—was duplicated in two 3R ohnologs, celf2a and celf2b, that were not retained as 2 copies in all teleost fish. The genomic context of the celf2a and celf2b paralogous regions clearly indicates a partition of the ancestral region found in spotted gar. The celf2a gene was retained in all species investigated, but the celf2b gene seems to have been lost in Otophysi or at least in D. rerio (Cypriniformes), Astyanax mexicanus (Characiformes), and Ictalurus punctatus (Siluriformes). celf2, CUGBP Elav-like family member 2; Chr 8, Chromosome 8; Lrpprc, leucine rich pentatricopeptide repeat containing; Ol-BSF, Oryzias latipes Bicoid Stability Factor; Ol-CUG-BP, Oryzias latipes CUG-binding protein. (TIF) [file pbio.3000185.s002.tif]

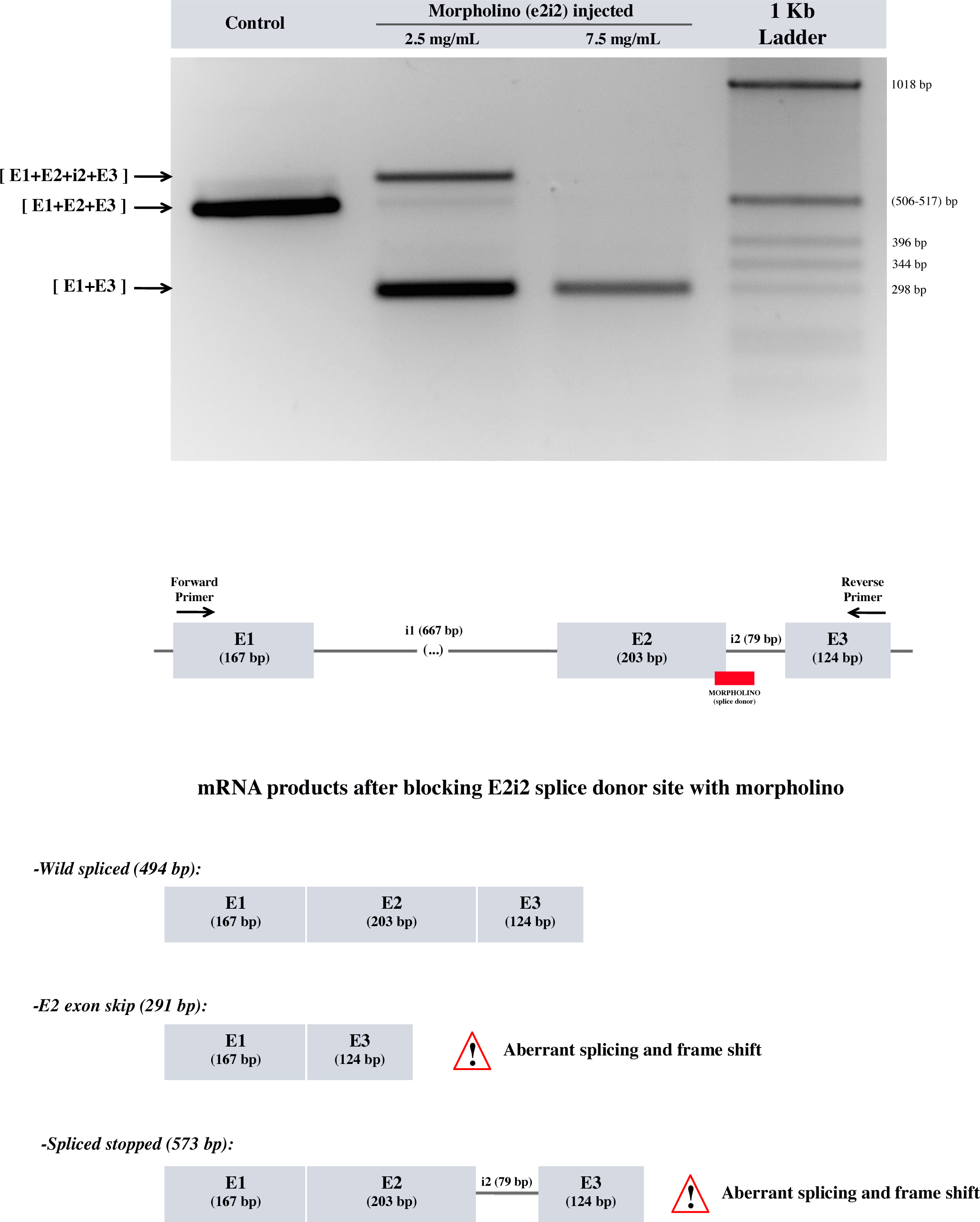

Supplement: S3 Fig — For in vivo transient down-regulation of Ol-bsf, a splice morpholino was designed to encompass the splice junction between exon 2 and intron 2 of the Ol-bsf gene in order to induce aberrant splicing and frame shit of the ORF. To show to what extend the splicing/activity of Ol-bsf was impacted, RT-PCR using exons 1, 2, and 3 spanning primers together with cDNAs from different stages of morpholino-injected embryos was achieved. E2, exon 2; i2, intron 2; Ol-BSF, Oryzias latipes Bicoid Stability Factor; RT-PCR, Reverse Transcription-Polymerase Chain Reaction. (TIF) [file pbio.3000185.s003.tif]

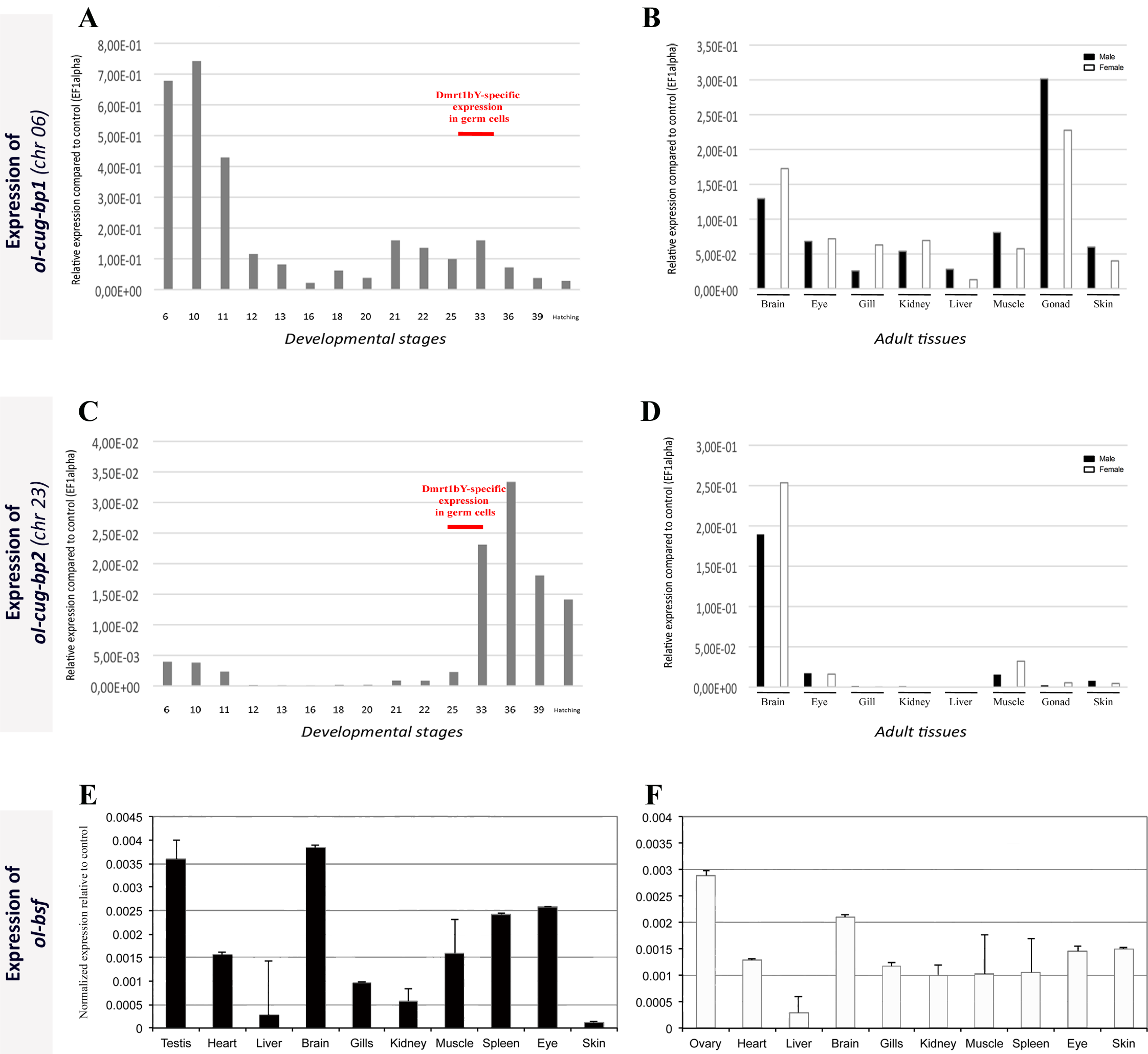

Supplement: S4 Fig — (A and C) During embryonic development, both Ol-cug-bp ohnologs are expressed in a complementary manner. Being likely maternally deposited the expression of Ol-cug-bp1 rapidly decreases after mid-blastula transition (stage 10) to remain virtually off up to hatching stage. On the other hand, low expression of Ol-cug-bp2 is detected until stage 25 and rapidly increases by stage 33. (B and D) In adult tissues, both Ol-cug-bp ohnologs are expressed in brain, muscles, and gonads; ol-cug-bp2 is additionally expressed in eyes and skin. Both ohnologs are higher expressed in male gonads than in female gonads. (E and F) In adult tissues, Ol-bsf is ubiquitously present although higher expression is observed in gonads of both sexes. Underlying data for (A to F) can be found in S1 Data. (TIF) [file pbio.3000185.s004.tif]

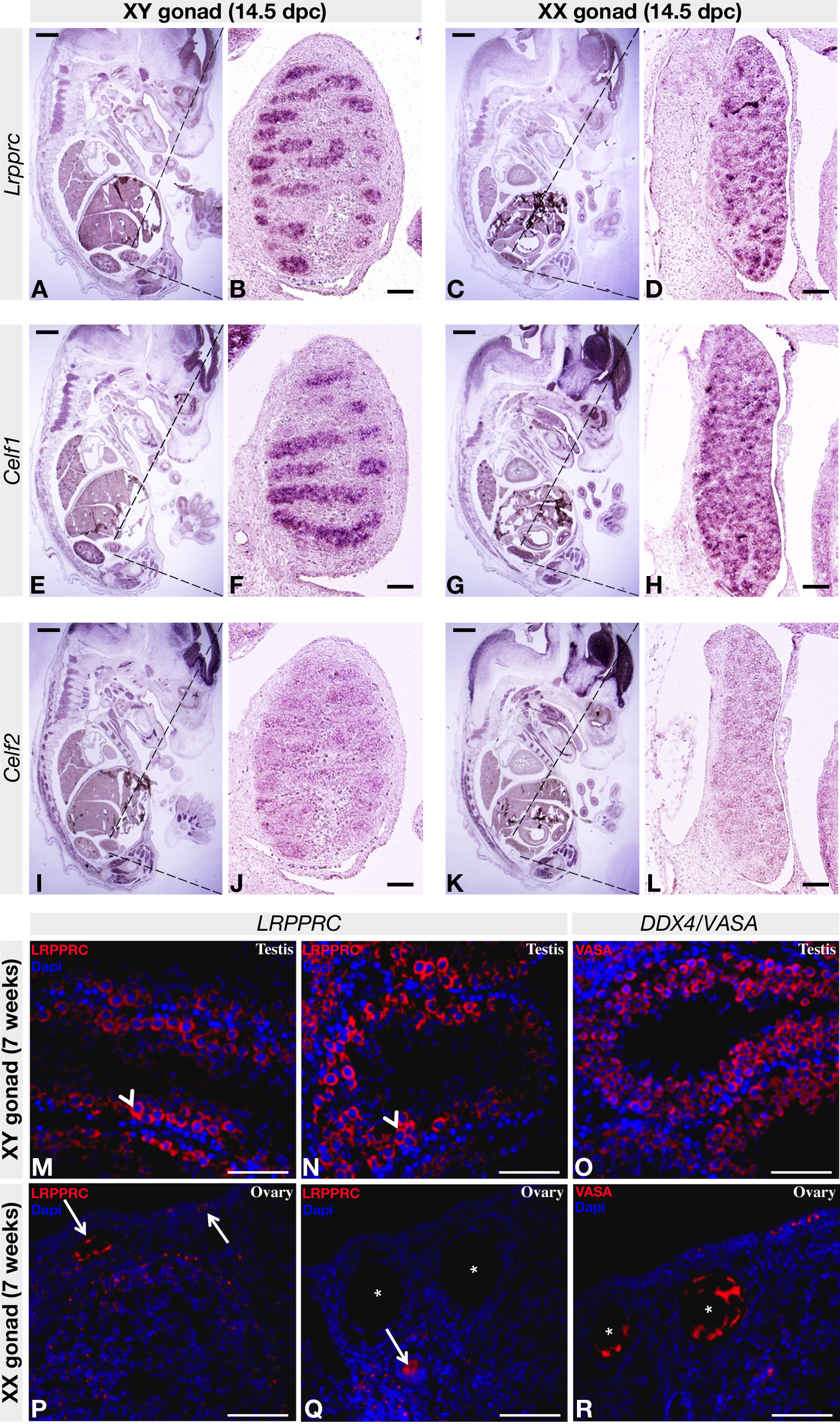

Supplement: S5 Fig — (A to H) ISHs on sagittal sections of 14.5 dpc mouse embryos showed expression of lrrprc (A to D) and celf1 (E to H) most likely in germ cells within testis cords (B and F) and germ cells within the ovary (D and H). In contrast, no celf2 expression was detected in developing gonads (I–L). However, celf2 expression was detected in other tissues, such as part of the brain and dorsal root ganglia. Scale bars: 1 mm for A, C, E, G, I, and K; 10 mm for B, D, F, H, J, and L. (M–R) Immunofluorescent detection of LRPPRC (M, N, P, Q) and DDX4/VASA (O, R) in adult mouse testes (M–O) and ovaries (P–R). In adult testes, lrpprc is expressed in one subpopulation of germ cells; compared lrpprc staining on (M) and (N) with vasa staining on (O) where most of the germ cells (except some spermatogonia) remain stained by vasa. According to the position of lrpprc-positive cells (arrowheads in M and N) in the seminiferous tubule (not basal and below round spermatids) and to the fact that lrpprc-positive germ cells are those with the largest nucleus, lrpprc-positive cells seem to be spermatocytes at the pachytene stage. In adult ovaries (P–R), lrpprc is mainly expressed into the oocytes of primordial, primary and young secondary follicles (see arrows on [P] and [Q]). Lrpprc staining disappears from the oocyte of secondary follicles that are clearly stained for vasa in (R) (compared stars in [Q] and [R]). Scale bars: 200 μm for M to R. dph, days post hatching; ISH, in situ hybridisation; LRPPRC, leucine rich pentatricopeptide repeat containing. (TIF) [file pbio.3000185.s005.tif]

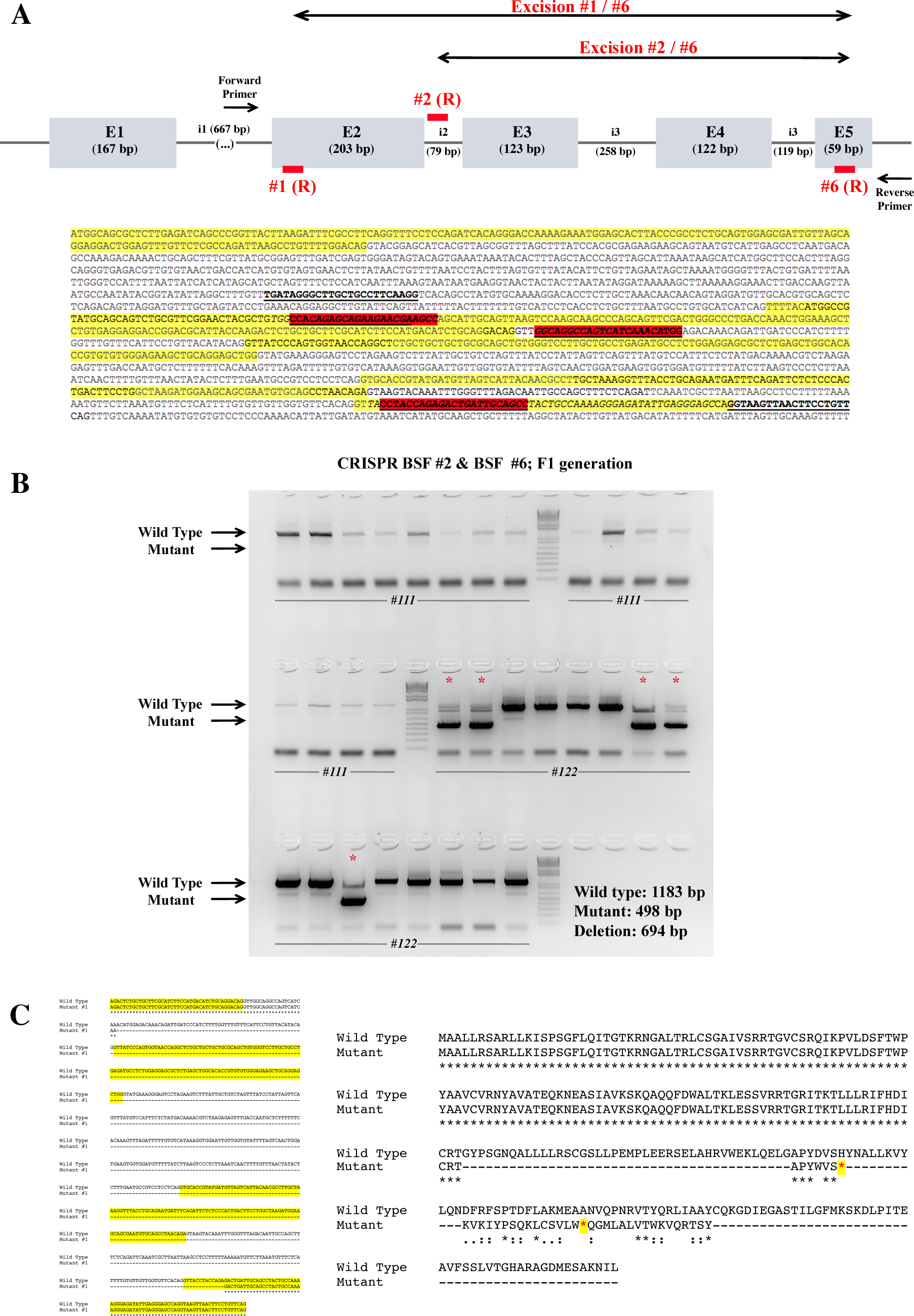

Supplement: S6 Fig — (A) Several guide RNA were designed in order to target different locations on the Ol-bsf gene (targets 1, 2, and 6). (B) After injection of different combinations of guide RNAs together with the Cas9 mRNA, putative edited fish were subjected to RT-PCR using primer sets flanking the cutting sites. Lines displaying deletions within the Ol-bsf gene (red stars) were kept for further investigations. (C) Deletions obtained within the Ol-bsf gene (left panel) result in a truncated translated Ol-bsf protein (right panel). CRISPR-Cas9, clustered regularly interspaced short palindromic repeats/CRISPR-associated protein 9; Ol-BSF, Oryzias latipes Bicoid Stability Factor; RT-PCR, Reverse Transcription- Polymerase Chain Reaction. (TIF) [file pbio.3000185.s006.tif]

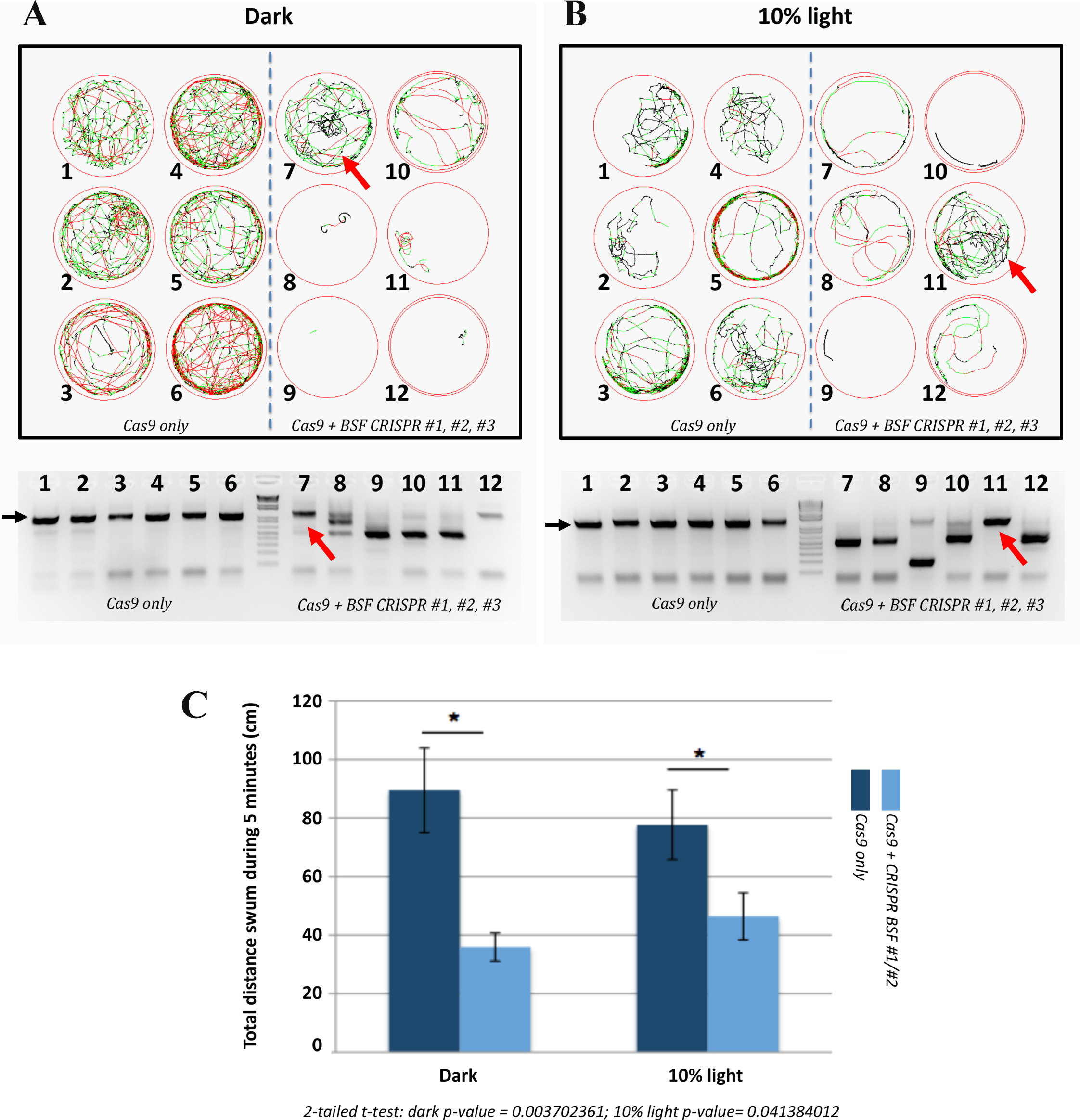

Supplement: S7 Fig — Locomotor activity (Ol-bsf mutants versus wild type) was determined at hatching stage (stage 39, 9 dpf) either under dark conditions (A) or with 10% light (approximately equal to 100 lux [panel B]) by measuring the total distance swum during a period of 5 minutes. (C) The total distance swum for each individual is equal to the sum of distances reached during inactivity, small and large movements. Bars and error bars indicate mean ± standard deviation. N = 12 for each condition. Underlying data for (C) can be found in S1 Data. dpf, days post fertilization; Ol-BSF, Oryzias latipes Bicoid Stability Factor. (TIF) [file pbio.3000185.s007.tif]

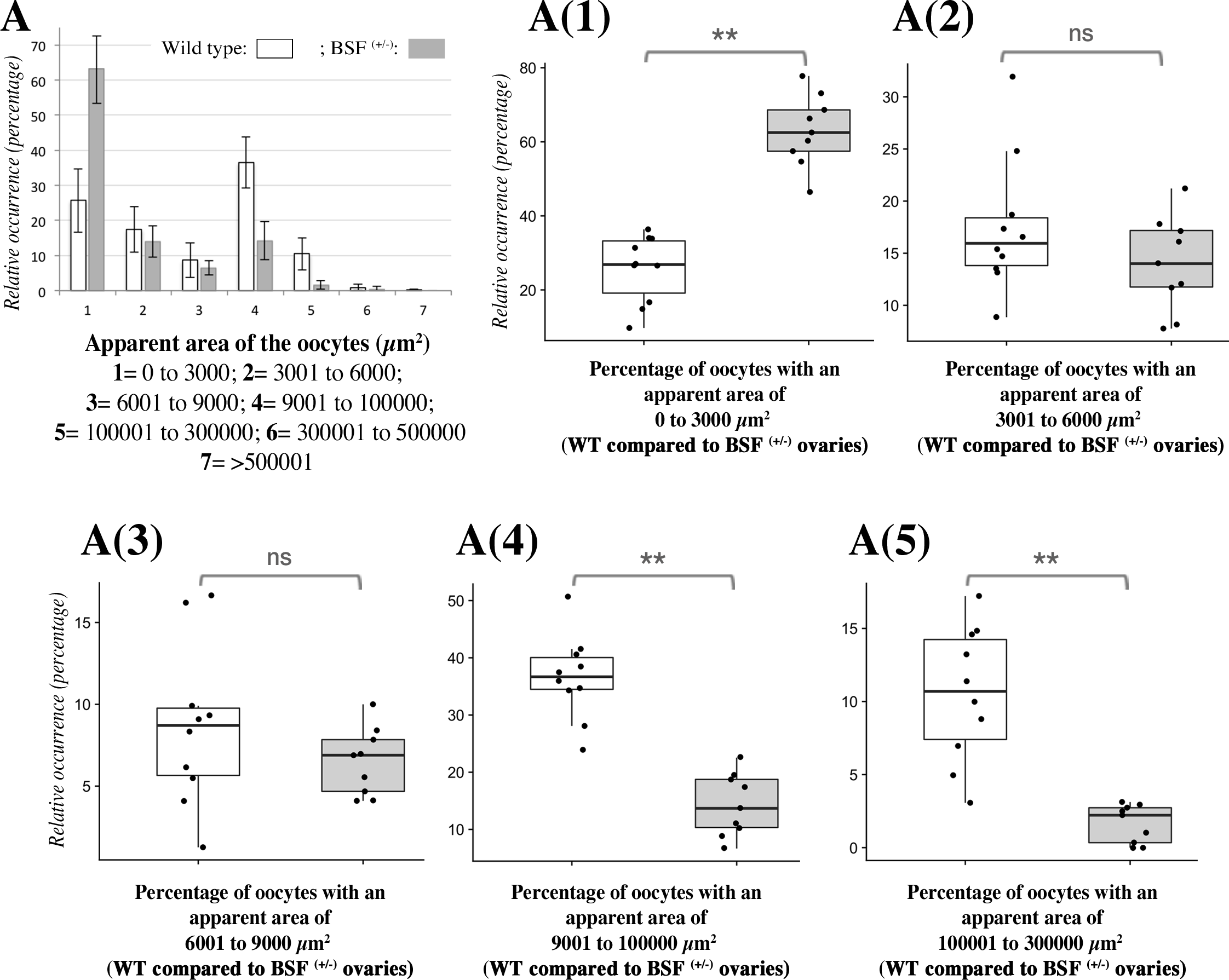

Supplement: S8 Fig — Morphological inspection of heterozygote mutant ovaries discloses a significant accumulation of small sized-oocytes compared to wild type (A) and (A1 to A5 for details and statistical analyses). (A) Overall size distribution of the oocytes in 9 wild-type and 9 Ol-BSF(+/−) adult ovaries. Each gonad (testes or ovaries) was sectioned through the mid-sagittal plan (see also Materials and methods). Underlying data for (A) can be found in S1 Data. Ol-BSF, Oryzias latipes Bicoid Stability Factor. (TIF) [file pbio.3000185.s008.tif]

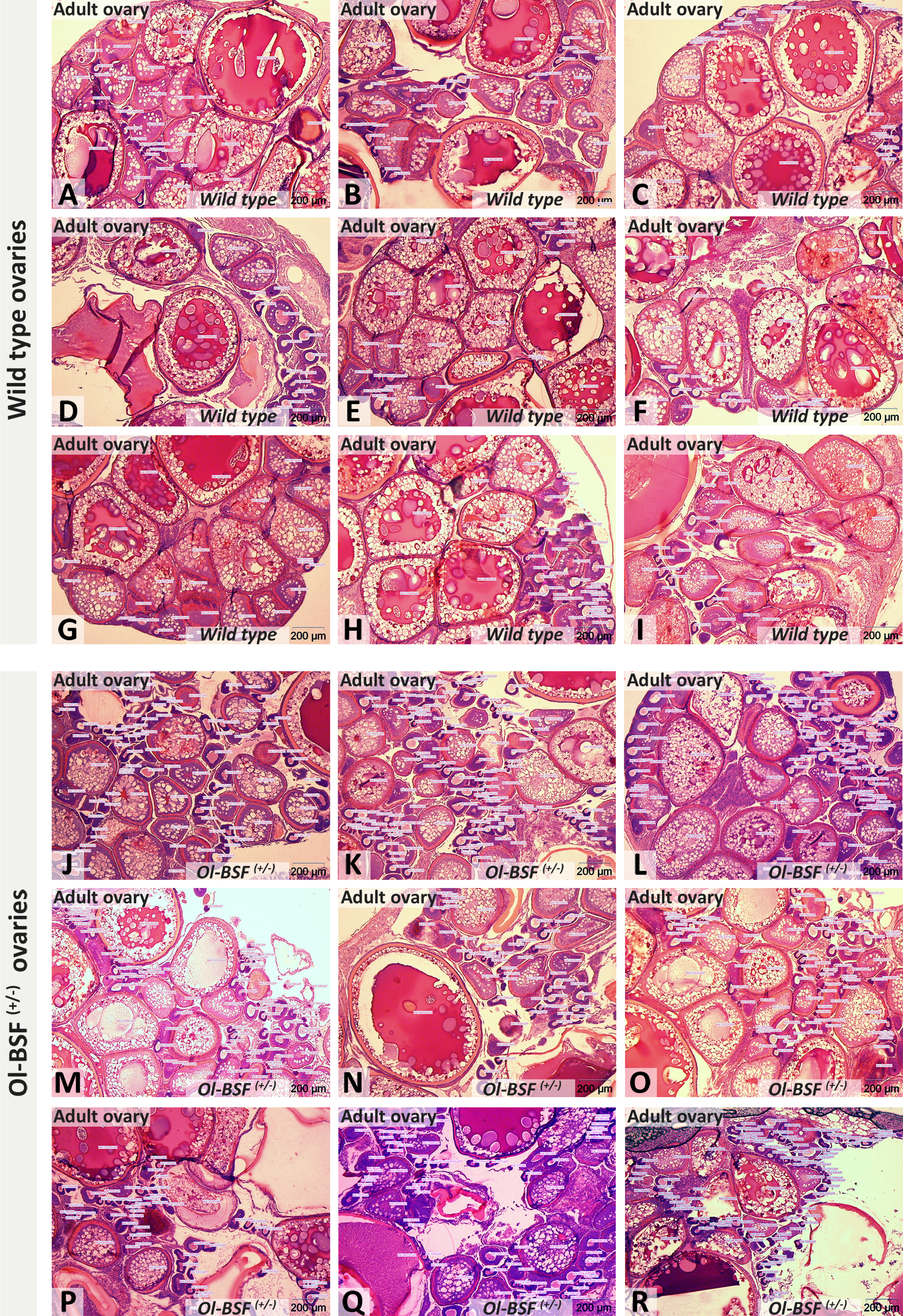

Supplement: S9 Fig — (A to R) Mid-sagittal sections of the ovaries utilized for counting the oocytes in S8 Fig. Each gonad (testes or ovaries) was sectioned through the mid-sagittal plan (see also Materials and methods). Underlying data for (A) can be found in S1 Data. Ol-BSF, Oryzias latipes Bicoid Stability Factor. (TIF) [file pbio.3000185.s009.tif]

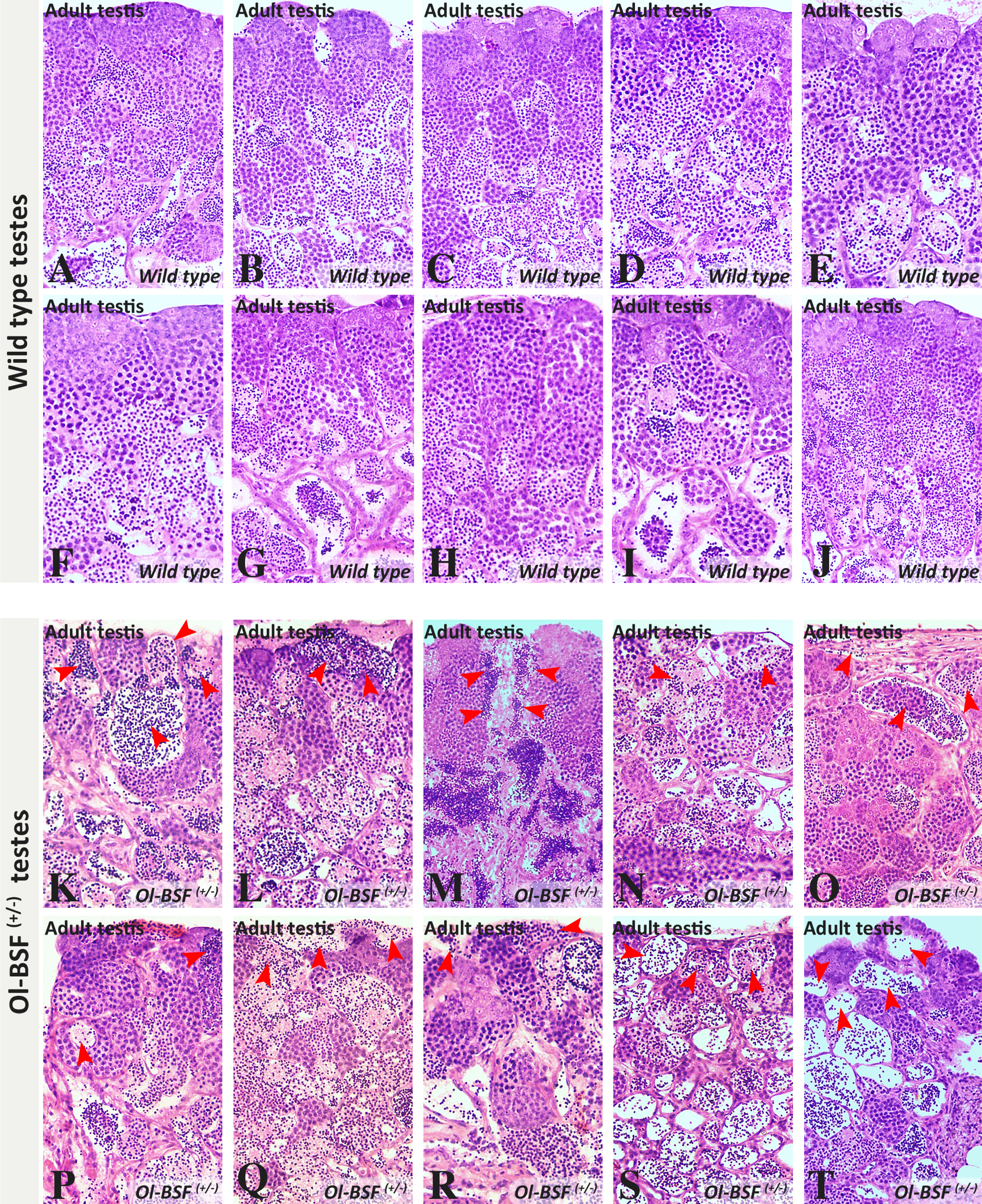

Supplement: S10 Fig — (A–J compared to K–T) Heterozygote mutant testes (A–J) exhibit a decreased number of spermatogonia with accumulation of type 2 spermatocytes, spermatids, and sperm within the most external layers of the seminiferous epithelium (arrowheads) compared to wild-type testes (K–T). Either 10 different wild-type (A–J) or Ol-bsf-deficient (K–T) testes were analysed. Mid-sagittal gonad sections were stained with haematoxylin–eosin–safran. Each gonad (testes or ovaries) was sectioned through the mid-sagittal plan (see also Material and methods). Ol-BSF, Oryzias latipes Bicoid Stability Factor. (TIF) [file pbio.3000185.s010.tif]

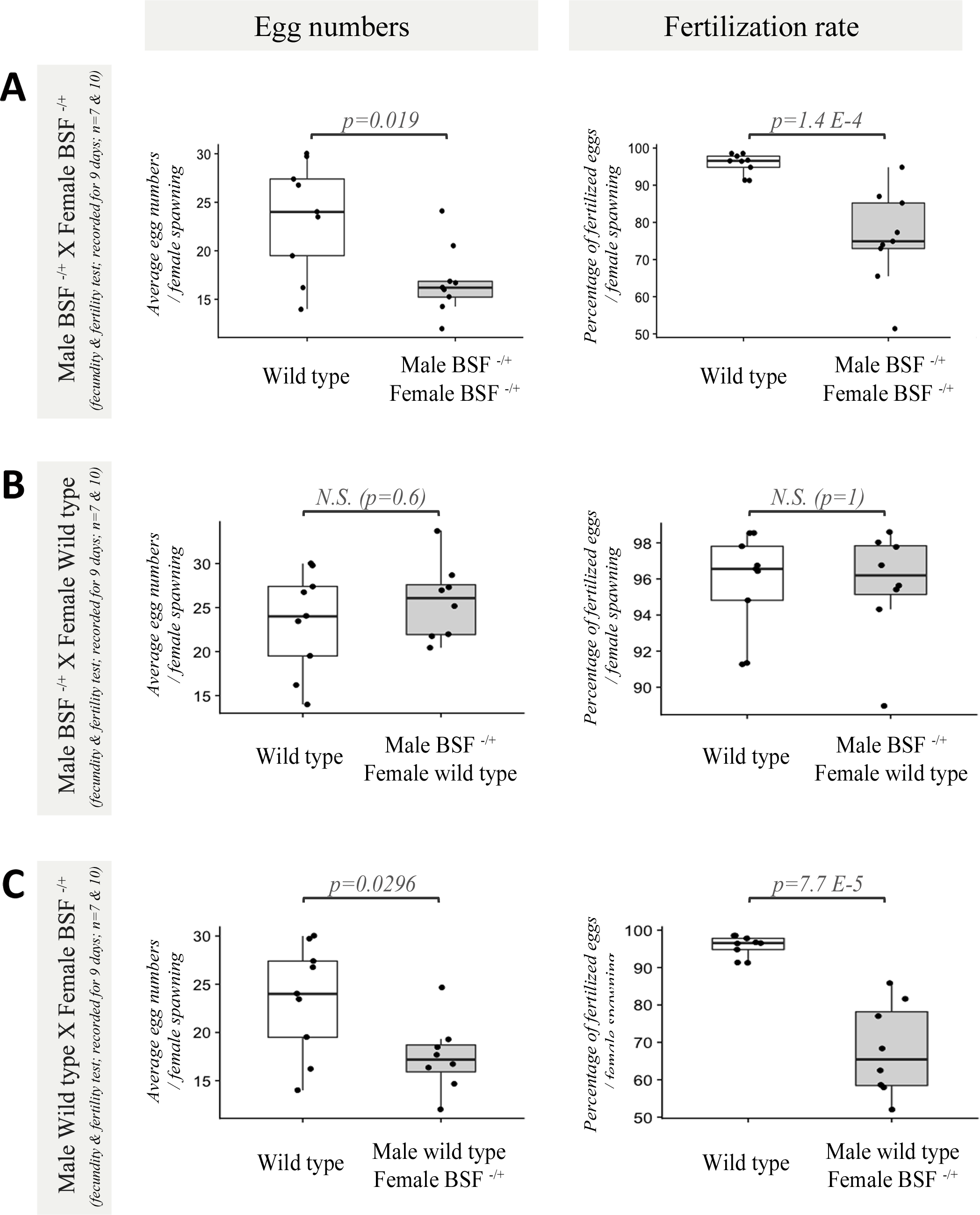

Supplement: S11 Fig — Egg numbers and fertilization rates were recorded over a period of 9 days for the following crosses: (A) male Ol-bsf (−/+) × female Ol-bsf (−/+); (B) male Ol-bsf (−/+) × female wild type; (C) male wild type × female Ol-bsf (−/+). Underlying data for (A to C) can be found in S1 Data. BSF, bicoid stability factor. (TIF) [file pbio.3000185.s011.tif]

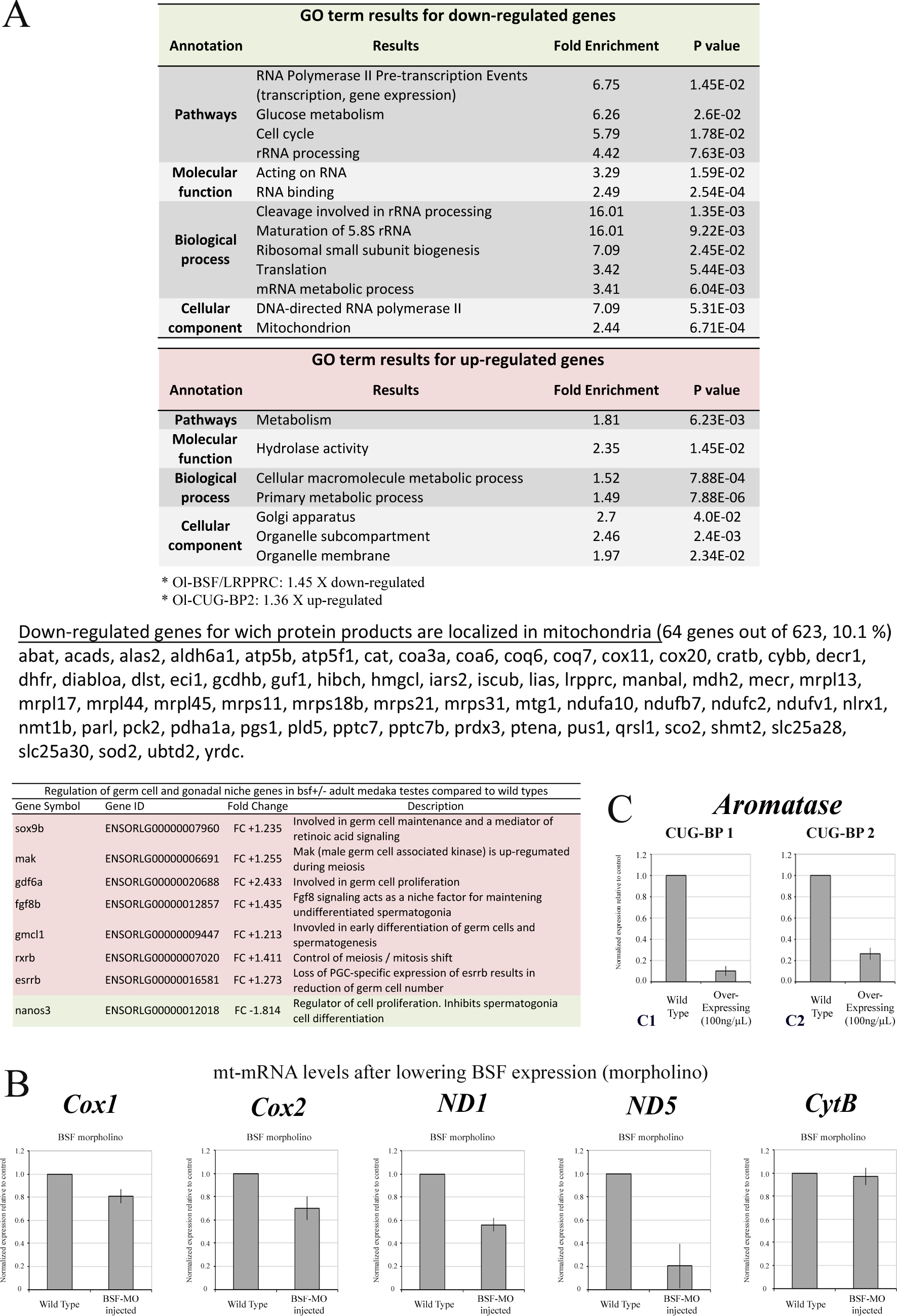

Supplement: S12 Fig — (A) Microarray. Adult testes of either bsf+/− or wild-type animals were subjected to microarray (see Materials and methods). GO term analysis reveals that in mutant testes partially depleted for the bsf gene, rRNA processing is particularly affected. Ol-bsf and Ol-cug-bp2 are down- and up-regulated, respectively, in mutant animals compared to wild type. Of note, and in accordance with the literature, a significant proportion (10.1%) of the down-regulated genes code for proteins localized in the mitochondria. Finally, supporting our observations that lowering ol-bsf transcription (morpholino injection in Fig 7E) resulted in up-modulation of germ cell number and that mutant gonads presented an increase of germ cells committing to gametogenesis (Fig 6), our microarray analysis reveals a general up-regulation of genes involved in germ cell proliferation or differentiation. (B) RNA levels of different mitochondrial genes (Cox1, Cox2, ND1, ND5, and CytB) were quantified by real-time PCR after BSF-morpholino injections and compared to wild type. Most of the mitochondrial genes are down-regulated when the level of Ol-bsf decreases. (C) Modulation of RNA levels of the cyp19a1 (aromatase) gene after overexpression of Ol-cugbp1 or Ol-cug-bp2. Underlying data for (B and C) can be found in S1 Data. GO, gene ontology. (TIFF) [file pbio.3000185.s012.tiff]
